# Diagnostic accuracy in the Swedish national patient register: a review including diagnoses in the outpatient register

**DOI:** 10.1007/s10654-025-01221-0

**Published:** 2025-03-27

**Authors:** Åsa H. Everhov, Thomas Frisell, Mehdi Osooli, Hannah L. Brooke, Hanne K. Carlsen, Karin Modig, Karl Mårild, Jonathan Lindström, Karin Sköldin, Mona Heurgren, Jonas F. Ludvigsson, Ola Olén

**Affiliations:** 1 Division of Clinical Epidemiology, Department of Medicine Solna, Karolinska Institutet, Stockholm, Sweden; 2 Department of Clinical Science and Education, Södersjukhuset, Karolinska Institutet, Stockholm, Sweden; 3 Medical Epidemiology, Department of Surgical Sciences, Uppsala University, Uppsala, Sweden; 4 Centre of Registers Västra Götaland, Gothenburg, Sweden; 5 Section of Psychiatry and Neurochemistry, Institute of Neuroscience and Physiology, Sahlgrenska Academy, University of Gothenburg, Gothenburg, Sweden; 6 Unit of Epidemiology, Institute of Environmental Medicine, Karolinska Institutet, Stockholm, Sweden; 7 Department of Pediatrics, Institute of Clinical Sciences, Sahlgrenska Academy, Gothenburg, Sweden; 8 Department of Pediatrics, Queen Silvia Children’s Hospital, Gothenburg, Sweden; 9 Department of Registers and Statistics, National Board of Health and Welfare, Stockholm, Sweden; 10 Department Medical Epidemiology and Biostatistics, Karolinska Institutet, Stockholm, Sweden; 11 Department of Pediatrics, Örebro University Hospital, Örebro University, Örebro, Sweden; 12 Department of Pediatric Gastroenterology and Nutrition, Sachs’ Children and Youth Hospital, Stockholm, Sweden; 13 Department of Surgery, Stockholm South General Hospital, 118 61 Stockholm, Sweden

**Keywords:** Classification of diseases, Epidemiology, Register, Validation studies, National patient register, Sweden, Register-based epidemiology, Administrative healthcare register

## Abstract

**Background:**

The Swedish National Patient Register (NPR) is an important source of data for epidemiological research. A review in 2010 described the validity of recorded diagnoses for inpatient care, but did not include specialised outpatient care.

**Method:**

Using systematic searches of medical literature databases (Embase, Medline), and reports from members of the Swedish Epidemiological Association, we aimed to identify all studies validating diagnoses and procedure codes in inpatient care since 2010 and all studies validating specialised outpatient care. In addition, we summarize findings from register validation work performed by the National Board of Health and Welfare.

**Results:**

The literature search and personal reports generated 3990 non-duplicate original studies, of which 89 were deemed relevant. Compared to data in patient charts (reference), the median positive predictive value (PPV) for diagnostic codes in the NPR was 84% (interquartile range 72–93%), but with clear differences between types of diagnoses. The median PPV for surgical procedures was 97% (86–99%). The median sensitivity of diagnoses and procedures compared to other registers and cohorts was 73% (45–80%). The completeness of the register has improved over time. Missingness originates mainly from underreporting of procedures performed by private healthcare providers, and for certain variables, e.g. medication codes.

**Conclusion:**

The NPR has good diagnostic accuracy for most diagnoses and very good for surgical procedures. The sensitivity is lower. Longitudinal comparisons of incidence or prevalence are affected by changes in completeness. Missingness is low, although it is higher among private healthcare providers and for specific variables such as drug administration.

**Supplementary Information:**

The online version contains supplementary material available at 10.1007/s10654-025-01221-0.

## Introduction

The Swedish healthcare system is tax-funded and universal. It is legislated by the state, which also provides guidelines and monitoring. The regions manage healthcare at the primary, secondary and tertiary levels, whereas other healthcare, e.g., nursing home care, is managed by the municipalities. Both public- and private-owned clinics operate at all levels.

The Swedish National Patient Register (NPR), managed by the National Board of Health and Welfare, includes data on hospitalisations in the “Inpatient register” with national coverage since 1987, and on specialised outpatient care in the “Outpatient register”, nationwide since 2001. The purpose of the NPR is to cover all hospitalizations and health care contacts of patients treated directly by physicians in specialized outpatient care [1]. For each healthcare contact, the NPR holds information on the patient's identity, diagnoses and procedures, and other medical and administrative information, making it an important source of data for epidemiological and clinical research. A detailed description of the register is in supplement “**Description of the National Patient Register**”.

A review of 132 studies validating diagnoses in the NPR recorded during hospitalisations was published in 2010 [2]. This review found that the positive predictive values (PPVs) differed between diagnoses but were generally 85–95%. Hospital-based outpatient registrations have been recorded in the NPR since 2001, but were not included in the previous review. The NPR has also undergone several changes since last reviewed. The aim of this study was to conduct an updated systematic review of studies validating the NPR, also including specialised outpatient care.

## Methods

The study was conducted following a predefined protocol.

### Inclusion criteria for the review of validation studies

Studies were considered eligible if they met all of the following criteria:

- Published research validating ≥ 1 code, e.g., diagnostic code (International Classification of Diseases, ICD), procedure code (Klassifikation av Vårdåtgärder, KVÅ), or medication code (Anatomical Therapeutic Chemical, ATC) recorded in the NPR
- Compared such codes in NPR data with an external reference standard, e.g., patient chart review, registers or independent cohorts
- Included estimates of sensitivity and/ or positive predictive value (PPV), or sufficient data to calculate such measures

### Exclusion criteria:

Studies were excluded from the review if they met the following criterion:

- Non-original studies including but not limited to literature reviews, systematic reviews, meta-analyses, commentaries, or editorials

All eligible, published articles were included, irrespective of study design. Validation studies on outpatient data were published from 2001 and onwards. For validation studies on inpatient data, the search was restricted to articles published on or after January 1st 2010, because validation studies of inpatient data before 2010 were included in the previous review [2].

### Search strategy

A librarian at Karolinska Institutet University Library conducted the literature search in Medline (Ovid) and Embase (embase.com). After the original search in June 2023, the search was last updated on 13 May 2024 by re-running the searches and de-duplicating against the previous results in EndNote [4]. For each search concept Medical Subject Headings (MeSH-terms) and free text terms were identified. The search was then translated to Embase syntax. No language restriction was applied. De-duplication was done in EndNote by comparing different fields [5] and by comparisons of Digital Object Identifiers.

Additionally, to ensure that we had identified all relevant studies, we emailed all members of the Swedish Epidemiological Association on June 8th, July 3rd, and Sep 5th 2023, and asked them to share published articles validating NPR data during the observation period. The full search strategies are available in the appendix (Supplementary Table 1 and 2).

### Selection process and data items

The literature search results were imported to Covidence (covidence.org) for title/abstract screening by a single reviewer (MO). The full texts of all potentially relevant references were independently reviewed by two co-authors according to the inclusion/exclusion criteria. The following data were extracted from the included full texts: first author, year, sample size, target study population/setting (single-centre, regional, national), period of observation, diagnosis/measure being validated, NPR code or NPR-based combinations of codes, henceforth called ‘NPR algorithm’, the reference standard data source to compare against the NPR, and the sensitivity and/or positive predictive value of code(s). The first author (ÅHE) compared the data extracted by the two reviewers; contradicting data was resolved through consensus or by a third reviewer.

### Statistics

If values for PPV or sensitivity were not reported, but could be calculated based on the data presented in the study in question, we estimated PPV as the number of patients fulfilling criteria for a diagnosis or procedure divided by the number of evaluated cases, i.e., the probability that the individual with the diagnostic code or algorithm truly had the disease in question. The sensitivity was calculated as the number of patients fulfilling criteria for a disease or procedure divided by the total number of patients with the disease/procedure in the cohort or register, i.e., what proportion of those that truly had the diagnosis/condition were captured in the NPR.

We calculated the median number of evaluated cases and the median values for PPV and sensitivity by grouping publications according to the data source to which the NPR was compared: *PPVs for diagnostic codes/algorithms compared to patient charts*, and *PPV and sensitivity compared to other registers or cohorts*.

## Results

The search generated 7,105 peer-reviewed articles. Members of the Swedish Epidemiological Association provided a list of 54 papers, of which 10 had not been captured by the initial search. Of the 3,990 non-duplicate original studies, 3,763 papers were excluded during abstract screening. From the remaining 227 articles, 138 were excluded after full-text screening, because they did not fulfil inclusion criteria (Supplementary Fig. 1).

We included 89 studies in the review, of which 65 described the validity of a diagnosis by comparing data in the NPR to patients’ charts (Supplementary Table 1), 17 studies compared data to quality registers or other cohorts (Supplementary Table 2), and 7 studies investigated the validity of non-diagnostic variables, such as medication codes and procedure codes (Supplementary Table 3).

Of the studies comparing register data to patient charts, the majority aimed primarily at validating NPR data. However, 20 studies included validation of NPR data as a sub-section of a publication with a different focus. Most studies used both inpatient and outpatient data for diagnosis definition, in some cases in combination with other nationwide administrative registers such as the Cause of Death Register [6–8], the Prescribed Drug Register [9–11] or the Swedish Cancer Register [12]. Some studies used only Inpatient Register data [7, 13–29], or only Outpatient Register data [29–32].

### PPV for diagnostic codes/algorithms compared to patient charts

The PPVs varied depending on type of diagnosis, diagnostic definition, e.g., number of listings, main or contributory diagnosis, number of cases assessed, and the chosen reference standard. The median number of evaluated cases was 166, interquartile range, IQR: 69–332. The median PPV was 84% (IQR 72–93%).

The lowest PPV at 18% was found for an algorithm trying to identify osteonecrosis of the jaw in postmenopausal women with osteoporosis in the absence of a specific diagnostic code for this condition [33]. The highest PPVs of 100% were reported for studies with few assessed cases: pneumoconiosis (7/7) [23], emotionally unstable personality disorder (26/26) [34], Wilson’s disease (26/26) [35] and “cardiopathy other than dilated or hypertrophic/obstructed” (20/20) [36].

In general, broader diagnostic groups generated higher PPVs than exact diagnoses; for example, inflammatory bowel disease had a PPV of 93%, while the inflammatory bowel disease subtype Crohn’s disease had a PPV of 72%, mostly because of misclassification with ulcerative colitis and inflammatory bowel disease -unclassified [37]. Similarly, any dementia had a higher PPV than Alzheimer’s dementia (96% and 64%, respectively) [38].

The diagnostic definitions varied, for example, some studies used only main diagnoses [21, 39–41], while others used main or first contributory diagnosis [21, 24, 39, 42–44]. Further, some studies required ≥ 2 listings on separate occasions [27, 32, 37, 41, 45–50], or listings from selected clinics [14, 31, 32].

**Fig. 1:**
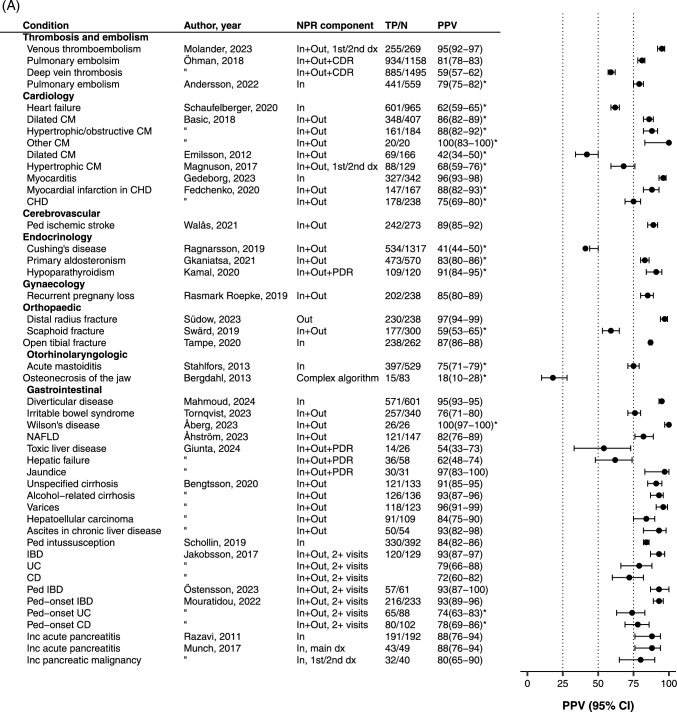

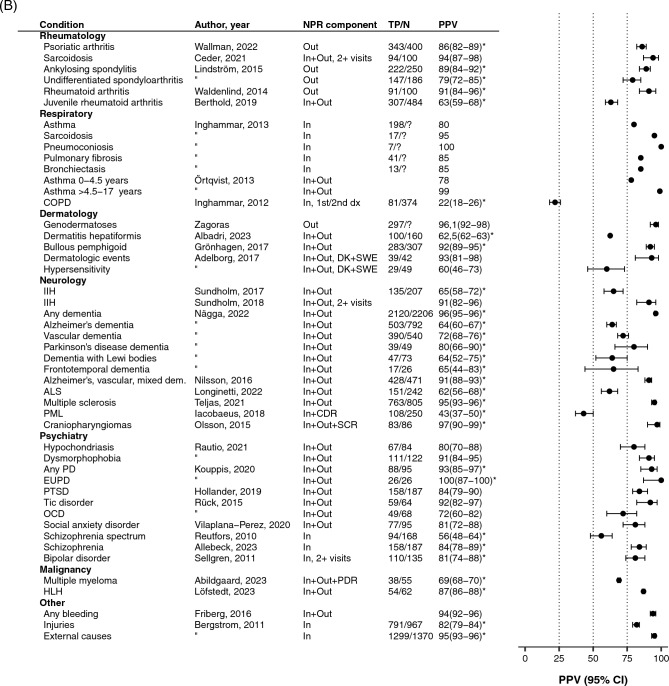
Positive predictive values (PPVs) with 95% confidence intervals (Cis) for diagnoses/conditions recorded in the Swedish National Patient Register compared to data in patient charts. CIs with * were calculated based on PPVs and number of evaluated cases. IBD, inflammatory bowel disease; CHD, congenital heart disease; UC, ulcerative colitis; CD, Crohn’s disease; COPD, chronic obstructive pulmonary disease; OCD, obsessive compulsive disorder; EUPD, emotionally unstable personality disorder; PML, progressive multifocal leukoencephalopathy; PTSD post-traumatic stress disorder; CM, cardiomyopathy; NAFLD, non-alcoholic fatty liver disease; PD, personality disorder; SCR, Swedish Cancer Register; ALS, amyotrophic lateral sclerosis; IIH, idiopathic intracranial hypertension; dx, diagnosis

In some studies, a smaller proportion of patients fulfilled strict predefined criteria, although most patients had a probable diagnosis, e.g., definite diagnosis of heart failure: PPV 62%, definite or probable diagnosis: 94% [14], chronic obstructive pulmonary disease: proven 21.7%; broader definition: 93% [24]. (Fig. 1).

### Sensitivity and PPV compared to other registers or cohorts

In studies comparing NPR data to quality registers, the median sensitivity, i.e., completeness, of the NPR was 73% (IQR 45–80%). The sensitivity was high for surgical procedures: 97% for humeral fractures [51], 88% for prostatectomies, and 86% for orchidectomies [52], and high for severe and sudden-onset diseases such as stroke at 91% [53]. The sensitivity was lower for psychiatric diseases. Out of all patients registered in National Quality Registers for eating disorders, 75% and 63% had a diagnosis of anorexia and bulimia nervosa in the NPR [54].

NPR-based definitions using combinations of more than one code generated lower PPVs. For instance, the sensitivity for Lyme borreliosis was 45% when comparing the number of patients who had the recommended combination of two different diagnostic codes in the NPR to the number of patients with positive cerebrospinal fluid–serum anti-Borrelia antibody index [55].

**Fig. 2:**
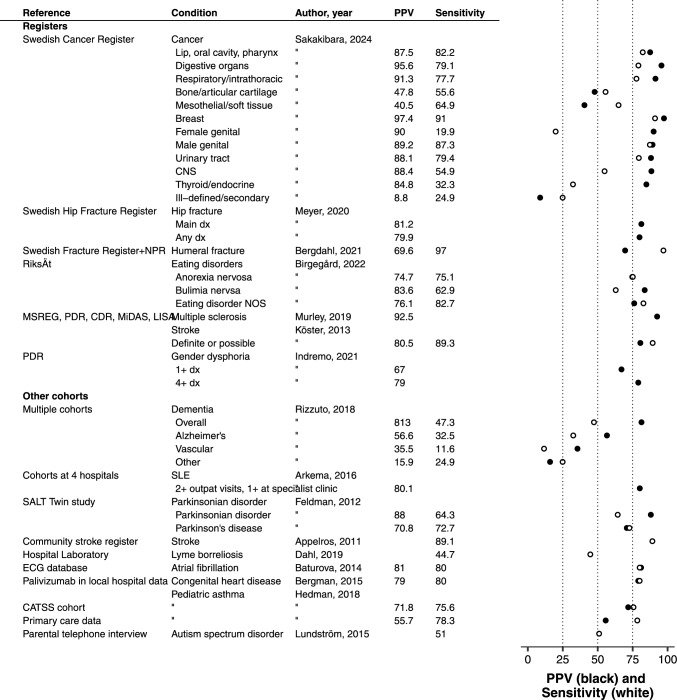
Sensitivity (empty circle) and positive predictive value (PPV, filled circle) for diagnoses/conditions recorded in the Swedish Patient Register compared to quality registers and other cohorts. NPR, National Patient Register; MSREG, Multiple Sclerosis Register; PDR, Prescribed Drug Register; CDR, Cause of Death Register; MiDAS, MikroData för Analys av Socialförsäkringen; RiksÄt, Nationellt kvalitetsregister för ätstörningsbehandling; dx, diagnosis; NOS, not otherwise specified; ECG, electrocardiogram; CATSS, The Child and Adolescent Twin Study in Sweden; SALT, Screening Across the Lifespan Twin Study; CNS, central nervous system

In studies reporting both sensitivity *and* PPV, the PPVs were higher than the estimated sensitivities for most cancer diagnoses [56], for dementia diagnoses [57], and for parkinsonian disorder, but with higher sensitivity than PPV for humeral fracture [51] and stroke [53]. The median PPV for all conditions was 72% (68–88) and the median sensitivity was 74% (47–80) when comparing to other registers or cohorts. (Fig. 2).

### Procedure codes

**Fig. 3:**
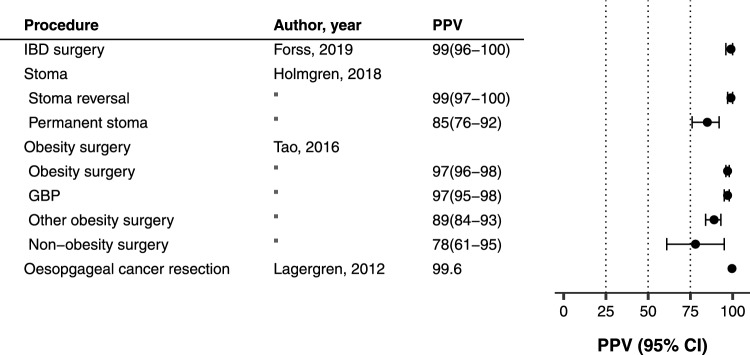
Positive predictive values (PPVs) for procedure codes recorded in the Swedish National Patient Register compared to data in patient charts. IBD, inflammatory bowel disease; GBP, gastric by-pass

Seven studies investigated procedure codes or medication codes. For surgical procedures, the PPVs were generally high: inflammatory bowel disease-related surgery 97% [58], stoma reversal 99% [59], obesity surgery 97% [60], oesophageal cancer resection surgery 99.6% [61]. Overall, the median PPV was 97%; IQR 86–99, Fig. 3. One study investigated medication codes for biological treatment in patients with inflammatory bowel disease comparing data on biologic treatment in the Prescribed Drug Register and the NPR with medical records. Based on the reported numbers, the sensitivity was calculated at 9.3% in the NPR, but with large differences between hospitals [62].

The results from the register validation performed by the National Board of Health and Welfare is described in Supplement “**Description of the National Patient Register**”.

## Discussion

The NPR is a valuable source of data for research purposes. It has nationwide coverage, little missing data, and overall good validity. This review identified 89 peer-reviewed articles comparing diagnostic codes or procedure codes in the NPR to an external data source. For evaluated diagnoses the median PPV was 84% compared to patient chart data, and the median sensitivity of diagnoses compared to other registers and cohorts was 74%. The median PPV for evaluated surgical procedures was 97%.

The accuracy and completenss of NPR data, as measured by PPV and sensitivity, varied depending on the codes used to define a diagnosis or procedure and the reference standard. The previous systematic review, which included only inpatient data [2], reported that the PPVs were generally 85–95% for most diagnoses. In our review, where most studies used codes from the entire NPR, i.e., both inpatient and outpatient data, the IQR of PPVs was 72–93%. The numbers are not entirely comparable, as the previous review did not calculate median or IQR, but PPVs appear lower in the present review. One explanation could be that, in the previous inpatient data review, many studies included only main discharge diagnoses, which increases the likelihood of a correct diagnosis, while studies in our review included all diagnoses (main and contributory, inpatient and outpatient). However, several validation studies including outpatient data reported excellent PPVs, either by requiring ≥ 2 diagnoses on separate occasions: inflammatory bowel disease 93% [37, 46, 47]; sarcoidosis 94% [48]; idiopathic intracranial hypertension 91% [49], only considering main or first contributory diagnoses: venous thromboembolism 95% [43], or diagnoses from selected clinics: rheumatoid arthritis 91% [32].

In NPR-based research, the choice of coding algorithm ultimately depends on the research question as employing stricter criteria will—in most cases—increase PPV but decrease sensitivity. For studies on mechanisms/underlying causes, a high PPV, i.e. low grade of misclassification of the outcome/disease, is probably advisable, whereas studies on incidence or prevalence need to use algorithms with reasonable sensitivity. For some conditions, the combination of the NPR and some other register source can increase diagnostic accuracy. For example, one of the included studies of this review required a dispensing of calcium/vitamin D supplementation in the Prescribed Drug Register for their definition of hypoparathyroidism [9].

The diagnostic method/technique of a condition itself can affect diagnostic certainty and thus the PPV observed. Diagnoses based largely on objective biomarkers, X-ray images or biopsy results likely have higher PPVs than diseases based on fulfilment of diagnostic criteria that are dependent on the physician’s judgment, as is the case for e.g. irritable bowel syndrome, a criteria diagnosis. With criteria-based diagnoses, it may also be more difficult to establish a definite diagnosis in a retrospective review due to a lack of sufficient data in patient charts.

No studies in this review pertained to diabetes mellitus type II, hypertension, or depression. Although these are highly prevalent in the population they are mostly managed in primary care, and thus not well covered by the NPR. Consequently, studies on such diseases are less often based on NPR data, unless the research question involves other conditions better covered by the NPR, such as cardiovascular disease.

Within the Nordic countries data from tax-funded universal healthcare can be used in research. Like the NPR, population-based nationwide registers of hospital care have existed in Finland since 1969, Iceland since 1999, Denmark since 1977, and Norway since 2008. These registers collect the same type of data as the NPR and allow individual-level linkage to other registers, and their validities are largely comparable to that of the NPR [63]. In a review of the Finnish Hospital Discharge Register most studies validated inpatient discharges from hospitals and health centres with PPVs between 75 and 99% [64]. The Danish National Patient Registry underwent an external validation in 2015, which found that PPVs of the reported diagnoses varied from below 15% to 100% [65]. A study on the Icelandic healthcare registries (the Hospital Discharge Registry and The Icelandic Register of Primary Healthcare Contacts) evaluated the validity of eight chronic diseases and reported an overall PPV of 93% in both registers combined [66]. The overall validity of nationwide healthcare registers in these countries is thus generally good, but similarly to the NPR, coverage and accuracy vary for individual diagnoses. Similarities in design and structure of national health registers across the Nordic countries enable collaborative research, particularly on rare events.

Healthcare register definitions of disease can vary from very simple to complex. Some examples of validated algorithms to identify inflammatory bowel disease in different countries are: Denmark: ≥ 1 diagnostic listing [67], Sweden: ≥ 2 diagnostic listings [37], Canada: ≥ 5 physician contacts or hospitalisations for an inflammatory bowel disease -related code [68], Israel: ≥ 5 diagnostic listings or ≥ 1 diagnostic listing combined with ≥ 3 purchases of inflammatory bowel disease related medications, or ≥ 3-month interval between the first and last purchase, or ≥ 2 purchases of steroids/5-aminosalicylic acid enemas [69]. The need for more complex algorithms in e.g., Canada and Israel can be explained by the contents of the database as well as healthcare organisation and reimbursement schemes: the Canadian and Israeli databases include both primary care and specialised care while the Swedish and Danish registers include only hospitalisations and specialised outpatient care. The Canadian algorithms were created based on billing claims in systems where coding for diseases such as inflammatory bowel disease was incentivised, as the physician was entitled to an extra financial compensation for seeing patients with chronic disease [47]. Similarly, financial compensation can affect coding patterns in countries with universal coverage, as diagnostic codes are used as the basis for reimbursement to the hospitals/clinics [70].

The overall aim of this review was to provide an overview of studies evaluating the NPR. We included studies with a diverse range of study designs and methodologies. This heterogeneity makes it challenging to apply a standardized quality assessment tool across all included studies. We did not perform a formal quality assessment, due to the large variability between studies regarding choice of register definition (from a single diagnostic code to complicated algorithms) and reference standard (from reviewers’ judgement to internationally accepted criteria).

Our conclusions may suffer from publication bias. Researchers presumably conduct validation studies with the aim to demonstrate that a certain register-based code is valid (and can be used in their research). The publication frequency of validation studies with unexpectedly poor results is unknown. Similarly, researchers may avoid performing validation studies when suspecting poor validity, e.g., due to known poor coverage in the NPR, conditions where treatments are not mainly prescribed by physicians, or where there is a need for complex algorithms. However, Supplementary Tables 3 to 5 present validation results for studies of a wide range of diagnoses, conditions, and procedures, of which several present mediocre or poor validity of register codes or algorithms. The data presented in this review include diseases that contribute to the largest burden to healthcare, are treated in hospital or specialised outpatients care, and have clear diagnostic criteria. Certain diagnostic groups are few or missing, e.g., infectious diseases, obstetric diseases, and cancer, most likely because of the availability of other register sources for these conditions.

Most studies included in this review identified patients with certain code/s recorded in the NPR and reviewed their charts to assess fulfilment of predefined diagnostic criteria, reporting the results as PPVs. PPV answers the question “What is the probability that the individual with this diagnostic code/algorithm truly has the disease in question?”. The sensitivity, i.e., what proportion of those that truly have the diagnosis/condition are captured in the NPR is more difficult to estimate and requires access to a screened population or a quality register with complete coverage. Furthermore, for conditions treated by prescribed medications, the addition of a drug dispensing may enhance diagnostic accuracy, however, few validation studies used the NPR in combination with data from the Prescribed Drug Register.

The NPR has some limitations with respect to its usefulness for research purposes: Its expansion over time can affect longitudinal comparisons. The outpatient part of the register has incomplete registration during the first years, particularly within the field of psychiatry. The proportion of underreporting, i.e., the systematic failure to capture health visits, from privately funded healthcare providers is unknown. Certain variables are notably incomplete, e.g., medication codes used as a supplement to drug administration is currently missing to such a degree that the official documentation advises researchers not to use it as only data source [3].

## Conclusion

PPVs and sensitivity vary depending on diagnosis, register definition, and the chosen reference standard. However, the NPR generally has good diagnostic accuracy with a median PPV of 84% for evaluated diagnoses when compared to patient chart data, and PPVs close to 100% for surgical procedures. Missingness is low, although it is higher among private healthcare providers and for specific variables such as drug administration. The coverage has increased over time, which can affect longitudinal comparisons.

## Supplementary Information

Below is the link to the electronic supplementary material.

Supplementary file1 (DOCX 191 KB)

Supplementary file2 (DOCX 420 KB)

## Data Availability

The current paper contains no original data but was based solely on secondary data.

## Abbreviations

NPR: National patient register
ICD: International classification of diseases
KVÅ: Klassifikation av Vårdåtgärder
ATC: Anatomical therapeutic chemical
PPV: Positive predictive value
