# Supplementary material for: Diagnostic accuracy in the Swedish national patient register: a review including diagnoses in the outpatient register
Source: Eur J Epidemiol. 2025 Mar 27;40(3):359–69. doi: 10.1007/s10654-025-01221-0 (PMC12137447; doi:10.1007/s10654-025-01221-0)
Supplement: Supplementary file 1 — Supplementary file1 (DOCX 191 KB) [file 10654_2025_1221_MOESM1_ESM.docx]

**Description of the Swedish National Patient Register (NPR)**

*Development of the NPR*

Inpatient healthcare data have been registered centrally in Sweden since 1964, and psychiatric care since 1973, with nationwide coverage since 1987. Day surgery was added in 1997, specialised outpatient care in 2001, medical procedures, i.e., KMÅ-codes in 2007, and data about compulsory psychiatric care in 2010. The annual number of reported healthcare contacts was just below 2 million in the year 2000, after which it increased sharply with the inclusion of specialised outpatient care. From 2013 and onwards, around 15 million contacts per year have been recorded, except for the year 2020, when outpatient visits declined due to the Coronavirus disease pandemic (COVID-19)(1).

**Figure 1**. Number of healthcare contacts in the National Patient Register from 1973 to 2023. Inpatient healthcare data have been registered with nationwide coverage since 1987. Day surgery was added in 1997 and specialised outpatient care in 2001

In 2015, the process for submitting information was changed, including more frequent reporting, the file structure was changed, and NPR started to collect data on waiting times at emergency departments. Since February 2017, abortions are reported to the register. Before that, data on abortions were anonymized in the register, effectively excluding them from most academic uses of the NPR (2).

*Reporting to the NPR*

The purpose of the NPR is to cover all hospitalizations and health care contacts of patients treated directly by physicians in specialized outpatient care, including those in both publicly funded and private healthcare services, while excluding any healthcare contacts solely provided by healthcare professionals other than physicians, such as midwives, nurses, dieticians, psychologists, or physiotherapists (1). However, from 2023 visits in psychiatry by other health professionals than physicians are reported to the register. All healthcare providers in Sweden are obliged by law to provide information to the NPR. From 2015 and onwards, data are submitted monthly and must include information on healthcare contacts during the three months preceding the reporting month. The information that is reported reflects the content of the medical records that healthcare providers must keep according to the Patient Data Act (2008:355). Most data are reported through the 21 Swedish regions, but some private healthcare providers report directly to the National Board of Health and Welfare (about 1.5% of inpatient and 6% of specialised outpatient care contacts). Alongside the clinical variables described below, the patient’s identity, year of birth, and sex are also reported to the NPR. For research purposes subject to ethical approval, NPR data can be linked to data from other Swedish registers or data sources by using the personal identity number (3). All records in the NPR revolve around the personal identity number.

*Variables in the NPR*

The NPR data variables relate to a specific healthcare contact, i.e., hospitalisation or physician visit, and include the care provider, the main diagnosis, relevant secondary diagnoses, procedures, and any external causes of injury. In Sweden, international classification of disease, ICD-10, coding was introduced in 1997 and procedures according to the Classification of Procedures (KVÅ) in 2007.

KVÅ uses the Swedish translation of the Nomesco Classification of Surgical Procedures combined with the Classification of Medical Procedures. Before 2007 only surgical procedures were submitted to the register. Anatomic Therapeutic Chemical (ATC) codes for administered drugs can be added to provide supplementary information to a diagnosis or KVÅ code.

The Diagnosis Related Groups (DRG) is a system for patient classification, performance, and reimbursement purposes. DRG weight is a measure of the treatment cost for an average patient in each DRG group, where higher weight equals higher costs.

A complete list of variables in the NPR is available at <https://www.socialstyrelsen.se/en/statistics-and-data/registers/national-patient-register/>.

**Register validation performed by the National Board of Health and Welfare**

The report *Production and quality of the National Patient Register* (2) describes the content, validation process, and estimated coverage of NPR. We summarize the most relevant points below.

*Completeness of the NPR*

The purpose of the NPR is to cover all hospitalisations and healthcare contacts by physicians in the specialised outpatient care in Sweden. The completeness, i.e., the proportion of recorded events in the covered population, is considered very high but is not possible to calculate in the absence of a reference standard. The municipalities and regions in Sweden present their own statistics on healthcare visits. These data are the basis for reimbursement and can be supposed to be complete. In comparison to their numbers, the completeness of the NPR has increased from 2005 and onwards. In 2020, the completeness was 96% for stroke compared with the National Stroke Register and 94% for myocardial infarction compared with SWEDEHEART/RIKS-HIA (2). While acute surgeries, almost exclusively performed in public healthcare, have a very high coverage in the NPR, types of surgeries that are performed in both public and private healthcare have a higher degree of missingness in NPR. E.g., the NPR captured 42% of treatments for varicose veins in 2018 (4), and about 80% of the operations registered in the Scandinavian Obesity Surgery Registry) (2). These observed missing visits and procedures pertain mostly to small to medium-sized private providers, and to procedures that are reimbursed by private insurance or paid by the patients themselves and not by public health insurance. (2)

*Missing variables in the NPR*

Of all registrations of hospitalisations in the NPR during the period 1987–2023, approximately 1.5% lacked a valid personal identity number and 1% had no main diagnosis. Within specialised outpatient care, approximately 1% of visits lacked a personal identity number and 12% were missing a main diagnosis during 2001–2023. The proportion of outpatient contacts missing a main diagnosis was substantial during the first year; 80% in psychiatric outpatient care and 30% in somatic outpatient care, but has continuously decreased, and in 2023 just under 3% of contacts lacked a main diagnosis (**Figure 2**). Lack of the main diagnosis is more common in outpatient care than in inpatient care, and more common in child and youth psychiatry than in adult psychiatry. For diagnoses of injury or poisoning, <5% of codes indicating the external cause are missing on the national level, but more often missing for some years and regions.

**Figure 2**. Proportion healthcare contacts (somatic/psychiatric hospitalisations/outpatient visits) missing a main diagnosis per year in the National Patient Register 1973 to 2023

Non-surgery procedures, i.e., KMÅ codes are generally underreported, and large regional differences occur, especially for procedures related to drug administration. For example, in 2021, the proportion hospitalisations with a code for drug administration varied from 5% to 89% between regions. Among hospitalisations with a code for drug administration, 68% lacked ATC code for type of administered drug (2) .

**References**

1. Ludvigsson JA-O. How Sweden approached the COVID-19 pandemic: Summary and commentary on the National Commission Inquiry. (1651-2227 (Electronic)).

2. Socialstyrelsen. Production and Quality of the National Patient Register 2023 [Available from: <https://www.socialstyrelsen.se/globalassets/sharepoint-dokument/dokument-webb/statistik/production-and-quality-of-the-patient-register.pdf>.

3. Ludvigsson JF, Otterblad-Olausson P, Pettersson BU, Ekbom A. The Swedish personal identity number: possibilities and pitfalls in healthcare and medical research. European journal of epidemiology. 2009;24(11):659-67.

4. Åhström H BL. Behandling av varicer i Sverige – underrapporteringen är stor. Lakartidningen. 2021;118(20218).
