# Supplementary material for: Diagnostic accuracy in the Swedish national patient register: a review including diagnoses in the outpatient register
Source: Eur J Epidemiol. 2025 Mar 27;40(3):359–69. doi: 10.1007/s10654-025-01221-0 (PMC12137447; doi:10.1007/s10654-025-01221-0)
Supplement: Supplementary file 2 — Supplementary file2 (DOCX 420 KB) [file 10654_2025_1221_MOESM2_ESM.docx]

**Supplement to:**

**Review of the Swedish national patient register – structure, evolution, and validation studies**

Everhov ÅH, Frisell T, Osooli M, Brooke HL, Carlsen HK, Modig K, Mårild K, Lindström J, Sköldin M, Heurgren M, Ludvigsson JF, Olén O

Table of Contents

[Supplementary Table 1. Ovid MEDLINE(R) search strategy 2](#_Toc176073159)

[Supplementary Table 2. Embase search strategy 3](#_Toc176073160)

[Supplementary Figure 1. Flow-chart of included studies 5](#_Toc176073161)

[Supplementary Table 3. Included studies that validated a diagnosis/condition through comparison with data in patient charts 6](#_Toc176073162)

[Supplemenatry Table 4. Sensitivity or positive predictive value (PPV) for variables in the National Patient Register (NPR), compared to other registers, cohorts, or databases 25](#_Toc176073163)

[Supplementary Table 5. Studies validating ATC and KVÅ-codes in the National Patient Register (NPR) 31](#_Toc176073164)

[References 33](#_Toc176073165)

# **Supplementary Table 1.** Ovid MEDLINE(R) search strategy

| Interface: Ovid MEDLINE(R) ALL  Date of Search: 13 May 2024  Number of hits: 3,748  Comment: In Ovid, two or more words are automatically searched as phrases; i.e. no quotation marks are needed | Field labels   - exp/ = exploded MeSH term - / = non exploded MeSH term - .ti,ab,kf. = title, abstract and author keywords - adjx = within x words, regardless of order - * = truncation of word for alternate endings |
| --- | --- |
| Database(s): **Ovid MEDLINE(R) ALL**1946 to May 10, 2024 Search Strategy:   \| **#** \| **Searches** \| **Results** \| \| --- \| --- \| --- \| \| 1 \| Registries/ \| 111266 \| \| 2 \| exp Medical records/ \| 161487 \| \| 3 \| (registry or registries or register? or population based).ti,ab,kf. \| 428984 \| \| 4 \| (medical record* or health record* or patient chart*).ti,ab,kf. \| 193957 \| \| 5 \| or/1-4 \| 760580 \| \| 6 \| Sweden/ \| 82939 \| \| 7 \| (sweden or swedish).ti,ab,kf. \| 89602 \| \| 8 \| 6 or 7 \| 122532 \| \| 9 \| Inpatients/ \| 31105 \| \| 10 \| Outpatients/ \| 22733 \| \| 11 \| exp Hospitals/ \| 326986 \| \| 12 \| exp Hospitalization/ \| 300916 \| \| 13 \| (inpatient* or hospital* or discharge* or outpatient* or patient*).ti,ab,kf. \| 9195581 \| \| 14 \| or/9-13 \| 9302279 \| \| 15 \| exp Evaluation studies as topic/ \| 1235212 \| \| 16 \| (Validation study or evaluation study).pt. \| 360136 \| \| 17 \| exp "Sensitivity and Specificity"/ \| 655825 \| \| 18 \| (validat* or validit* or predictive value* or evaluat* or sensitivity or specificity).ti,ab,kf. \| 6322301 \| \| 19 \| or/15-18 \| 7286775 \| \| 20 \| 5 and 8 and 14 and 19 \| 4049 \| \| 21 \| limit 20 to yr="2001 -Current" \| 3748 \| | |

# **Supplementary Table 2**. Embase search strategy

| Interface: embase.com  Date of Search: 13 May 2024  Number of hits: 3,357  Comment: Emtree is the controlled vocabulary in Embase | Field labels   - /exp = exploded Emtree term - /de = non exploded Emtree term - ti,ab,kw = title, abstract and author keywords - NEAR/x = within x words, regardless of order - * = truncation of word for alternate endings |
| --- | --- |
| \| No. \| Query \| Results \| \| --- \| --- \| --- \| \| #17 \| #15 NOT #16 \| 3357 \| \| #16 \| #4 AND #7 AND #10 AND #13 AND [2001-2023]/py AND [conference abstract]/lim \| 1947 \| \| #15 \| #4 AND #7 AND #10 AND #13 AND [2001-2024]/py \| 5304 \| \| #14 \| #4 AND #7 AND #10 AND #13 \| 5557 \| \| #13 \| #11 OR #12 \| 8864556 \| \| #12 \| validat*:ti,ab,kw OR validit*:ti,ab,kw OR 'predictive value*':ti,ab,kw OR evaluat*:ti,ab,kw OR sensitivity:ti,ab,kw OR specificity:ti,ab,kw \| 8656858 \| \| #11 \| 'evaluation study'/de OR 'sensitivity and specificity'/de OR 'validation study'/de \| 819096 \| \| #10 \| inpatient*:ti,ab,kw OR hospital*:ti,ab,kw OR discharge*:ti,ab,kw OR outpatient*:ti,ab,kw OR patient*:ti,ab,kw \| 13424249 \| \| #9 \| inpatient*:ti,ab,kw OR hospital*:ti,ab,kw OR discharge*:ti,ab,kw OR outpatient*:ti,ab,kw OR patient*:ti,ab,kw \| 13424249 \| \| #8 \| 'hospital patient'/exp OR 'outpatient'/exp OR 'hospital'/exp OR 'hospitalization'/de \| 2193755 \| \| #7 \| #5 OR #6 \| 147067 \| \| #6 \| sweden:ti,ab,kw OR swedish:ti,ab,kw \| 117264 \| \| #5 \| 'sweden'/exp \| 97016 \| \| #4 \| #1 OR #2 OR #3 \| 1116250 \| \| #3 \| 'medical record*':ti,ab,kw OR 'health record*':ti,ab,kw OR 'patient chart*':ti,ab,kw \| 326068 \| \| #2 \| registry:ti,ab,kw OR registries:ti,ab,kw OR register$:ti,ab,kw OR 'population based':ti,ab,kw \| 632600 \| \| #1 \| 'register'/exp OR 'medical record'/exp \| 541118 \| | |

# **Supplementary Figure 1**. Flow-chart of included studies

# **Supplementary Table 3**. Included studies that validated a diagnosis/condition through comparison with data in patient charts

| **Author, year** | **Setting (observation)** | **NPR data part** | **Diagnosis/Condition** | **Definition** | | **Reference standard** | **PPV (%)** |
| --- | --- | --- | --- | --- | --- | --- | --- |
|  |  |  |  | **ICD 8/9** | **ICD 10** |  |  |
| **Thrombosis and embolism** |  |  |  |  |  |  |  |
| Molander, 2023 (1) | Regional  (2009-2018) | **NPR** | Venous thromboembolism (VTE)  Pulmonary embolism (PE)  Deep venous thrombosis (DVT)  Superficial venous thrombosis (SVT) | - | Patients with rheumatoid arthritis with a main or first contributory diagnosis:  PE: I26  DVT/SVT: I80, I81, I82 | PE: by computed tomography (CT) scan or scintigraphy.  DVT: by ultrasound, CT scan, or phlebography.  SVT: ultrasound or recorded by the treating physician. | VTE: 255/269=95 (92–97) |
| Öhman, 2018 (2) | Regional  (1985-2014) | NPR + CDR | Pulmonary embolism (PE)  Deep vein thrombosis (DVT) | Patients 30-60 years with a first incident diagnosis, NPR + CDR:  PE  ICD-8: 450.00, 450.01, 450.03, 450.09, 673.98  ICD-9: 415B, 673C  DVT  ICD-8: 451.98, 451.99, 671.01, 671.02  ICD-9: 451B, 451C, 451W, 451X, 671D, 671E | Patients 30-60 years with a first incident diagnosis, NPR + CDR:  PE  I26.0, I26.9, O88.2  DVT  I80.1, I80.2, I80.3, I80.8, I80.9, O22.3, O87.1 | PE: CT, pulmonary angiography, MRI, ventilation-perfusion lung scan, or autopsy.  DVT: CT, venography, ultrasonography, MRI, or autopsy. | DVT or PE: 1771/2450=72.3 (70.3-74.1)  PE 934/1158 = 80.7 (78.4-82.9)    DVT 885/1495 = 59.2 (56.7-61.7) |
| Andersson, 2022 (3) | Two regions/ Multicentre (2005) | Inpatient register | Pulmonary embolism (PE) | - | Inpatient register: I26.0-I26.9 | Imaging (CT angiography or ventilation‐perfusion [V/Q] scintigraphy) or autopsy | 441/559=79 |
| **Cardiology** |  |  |  |  |  |  |  |
| Schaufelberger, 2020 (4) | Single-center  (2000-2012) | Inpatient register | Heart failure (HF) | - | Main or contributory diagnosis, internal medicine or cardiology clinics, inpatient register: I50 | ESC criteria | Definite: 601/965= 62.3  Probable: 310/965= 32.1 |
| Basic, 2018 (5) | Multi-center  (1989-2009, every 5th year) | **NPR** | Dilated cardiomyopathy (DCM)  Hypertrophic cardiomyopathy (HCM)  Obstructive hypertrophic cardiomyopathy (OHCM)  Other cardiomyopathy (OC) | NPR (ICD-9): 425A, 425B, 425D, 425E, 425F, 425H, 425W, 425X, 674W, 429D | NPR: I42.0, I42.1, I42.2, I42.3, I42.4, I42.5, I42.6, I42.7, I42.8, I42.9, I43, O90.3, O90.4, I51.7 | ESC criteria | DCM: 348/407=85.5  HCM and OHCM: 161/184=87.5  OC: 20/20=100 |
| Emilsson, 2012 (6) | Nationwide  (2006-2008) | **NPR** | Dilated cardiomyopathy (DCM) | Patients with coaeliac disease and reference individuals  (ICD-9): 42.5E | Patients with caeliac disease and reference individuals: I42.0 | (1) positive echocardiographic examination (ejection fraction <40% and left ventricular diastolic diameter >32 mm/m2 of the body volume), (2) a coronary angiography without >50% stenosis, and (3) no record of other causes of heart failure and cardiomyopathy | 69/166=42.0 |
| Magnusson, 2017 (7) | Regional  (2006-2016) | **NPR** | Hypertrophic cardiomyopathy | - | First or secondary diagnosis registered in Cyklop (the data source for NPR)  Obstructive HCM: I42.1  Other HCM: I42.2 | ESC guidelines | 88/129= 68.2 |
| Gedeborg, 2023 (8) | Nationwide  (2020-2021) | Inpatient | Myocarditis | - | Patients <40 years, inpatient register: I400, I401, I408, I409, I411, I418, I514 | Brighton Collaboration diagnosis criteria | 327/342=96 (93-98) |
| Fedchenko, 2020 (9) | Nationwide  (1970-2015) | **NPR** | Myocardial infarction (MI) | Patients with congenital heart disease (CHD), NPR:  CHD  ICD-8: 746-746.99; 747–747.59  ICD-9: 745A, 745B, 745C, 745D, 747E  MI  ICD-8/9: 410 | Patients with congenital heart disease (CHD), NPR:  CHD  Q20, Q21, Q22, Q23, Q24, Q25, Q26 (Excluded: Q26.5, Q26.6)  MI: I21 | Fourth Universal Definition of Myocardial Infarction (2018) | CHD: 178/238=75  MI: 147/167=88  in patients with confirmed CHD |
| **Cerebrovascular** |  |  |  |  |  |  |  |
| Walås, 2021 (10) | Nationwide  (1969-2016) | **NPR** | Ischemic stroke | Patients <18 years, NPR (ICD- 8/9): 433, 434, 436 | Patients <18 years of age, NPR: I63, I64 | Stroke: radiology (MRI, CT or angiography); Probable stroke: Clinical course, treatment and judgement | 242/273=89 (85-92) |
| **Endocrinology** |  |  |  |  |  |  |  |
| Ragnarsson, 2019 (11) | Nationwide  (1987-2013) | **NPR** | Cushing’s disease (CD)  Cushing’s syndrome (CS) | NPR (ICD-9): 255A and/or 227D | NPR: 225A, E240+D352, E249+D352 | Clinical, biochemical, imaging, and histopathological data | Confirmed CD: 534/1317=41  Other forms of CS: 156/1317=12  Probable CD: 41/1317= 3 |
| Gkaniatsa, 2021 (12) | Regional  (1987-2016) | **NPR** | Primary aldosteronism | NPR (ICD-9): 255.B | NPR: E260 | (1) Diagnostic test confirming PA; (2) High plasma aldosterone concentration, low or suppressed renin, spontaneous hypokalemia; or (3) patients treated medically or surgically for PA | Confirmed: 473/570=83  Probable: 17/570= 3 |
| Kamal, 2020 (13) | Multicenter  (2004-2016) | **NPR +PDR** | Hypoparathyroidism | - | NPR and PDR: Hypoparathyroidism E20.0, E20.1, E20.8, E20.9, E89.2, D82.1, E31.0)  Related disorders E21.4, E31.8, E31.9, E83.5 (Excluded: E21.4, E31.8, E31.9, E83.5)  AND  on treatment with Calcium/ Vitamin D/Active vitamin D/ Teriparatide | Prespecified protocol | 109/120=91 |
| **Gynaecology** |  |  |  |  |  |  |  |
| Rasmark Roepke, 2019 (14) | Nationwide  (2003-2012) | **NPR** | Recurrent pregnancy loss | - | ≥3 consecutive registrations 90 days apart before 22 gestational weeks, NPR: N96.9, O26.2 | Ultrasound examination or diagnosis based on a positive hCG-test | 202/238=85 (80–89) |
| **Orthopaedics** |  |  |  |  |  |  |  |
| Swärd, 2019 (15) | Nationwide  (2006-2015) | **NPR** | Scaphoid fractures | - | NPR: S62.00, S62.01 | Plain radiographs, magnetic resonance imaging (MRI) scans, computer tomography scans and skeletal scintigraphy images | 177/300=59 |
| Tampe, 2020 (16) | Nationwide  (2007-2016) | Inpatient register | Open tibial fracture | - | Inpatient register: S82.11, S82.21, S82.31 | Consultant orthopedic surgeon’s judgement | 238/262=87 (86-88)  (2515/2845 * 0.86 + 330/2845 * 0.97 )  Moderate injuries: 117/136=86  Severe injuries: 121/126=96 |
| Südow, 2023 (17) | Nationwide  (2001-2015) | Only outpatient or only inpatient register | Distal radius fracture in adults (≥18 years) |  | S52.5  S52.6  D52.5 or S52.6 and NCI/NDJ29-99 | radiology report, radiograph or medical records | Outpatient:  230/238=97 (94-99)  217/236=92 (88-95)  236/236=100 (99-100)  Inpatient:  233/238=98 (95-99)  229/240=95 (92-98)  /236=96 (93-98) |
| **Otorhinolaryngologic** |  |  |  |  |  |  |  |
| Stahlfors, 2013 (18) | Multi-center  (1993-2007) | Inpatient register | Acute mastoiditis | Inpatient register: 383A | Inpatient register: H700 | Clinical signs of ongoing or recent acute mastoiditid and at least two (of) retroauricular signs and/or signs of an ear canal abscess. | 397/529=75 |
| Bergdahl, 2013 (19) | Nationwide  (2005-2009) | **NPR** | Osteonecrosis of the jaw (ONJ) in postmenopausal women women with osteoporosis | - | NPR: K04.6, K10.2, K10.3, M87.0, M87.1, M87.2, M87.3, M87.8, M87.9 | American Association of Oral and Maxillofacial Surgeons criteria | 15/83=18 (10-28) |
| **Gastrointestinal** |  |  |  |  |  |  |  |
| Mahmood, 2024 (20) | Nationwide (2002 and 2010) | **Inpatient** | Diverticular disease | - | K572-K579  Patients were excluded prior to, and up to 2 years after IBD, Celiac disease, IBS, colorectal and anal cancer | Computed tomography/x-ray of the colon/endoscopy*/*pain in the left lower abdominal pain, findings of elevated C-reactive protein combined with lack of vomiting*/*surgical findings and pathology report | 571/601=95 (93-95) |
| Tornkvist, 2023 (21) | Nationwide  (2005, 2010) | **NPR** | Irritable Bowel Syndrome (IBS) | - | Primary diagnosis in NPR: K58.0, K58.9  Patients with 17 differential diagnoses +/- 6 months were excluded. Also patients with *ever*-diagnosis of celiac disease/IBD/non-infective gastroenteritis-colitis were excluded. | Rome II critieria (2005); Rome III criteria (2010) | Correct diagnosis: 257/340=75.6 (70.8-79.9)  Correct+*probable* diagnosis: 80.3 (75.7–84.2)  IBS subtype: 61.5 (55.2–66.5) |
| Åberg, 2023 (22) | Single-center  (2002-2020) | **NPR** | Wilson’s disease |  | E83.0B | Not reported | 26/26=100 |
| Giunta, 2024 (23) | Nationwide  (2005-2016) | **NPR+PDR** | Toxic liver disease  Hepatic failure  Jaundice |  | ATC-code J02A in PDR, representing ≥7 DDD + ≥1 code for:  Toxic liver disease: K71.0, K71.1, K71.2, K71.6, K71.8, K71.9 Hepatic failure: K72.0, K72.9 Jaundice: R17  as a primary or secondary diagnosis | Toxic liver disease: modified Roussel-Uclaf Causality Assessment Method (RUCAM)  Hepatic failure:  coagulopathy with INR > 1.5 and liver encephalopathy OR  coagulopathy without ascites OR liver dysfunction with ascites  Jaundice: bilirubin > 80 μmol/L, or <80 and >35 plus a clinical findings of jaundice | 14/26=53.8 (33.4–73.4)  36/58=62.1 (48.4–74.5)  30/31=96.8 (83.3–99.9) |
| Åström, 2023 (24) | Single-center  (2015-2021) | **NPR** | Non-alcoholic fatty liver disease | - | NPR: K76.0 | Positive evidence upon pathology, radiology, or if the diagnosis was made by a consultant in hepatology | 121/147 = 82 (76–89) |
| Bengtsson, 2020 (25) | Nationwide  (2000-2016) | **NPR** | Cirrhosis | - | NPR:  Other and unspecified cirrhosis of liver: K74.6  Alcohol-related: K70.3  Oesophageal varices: I85.0, I85.9  Ascites: R18.9  Hepatocellular carcinoma (HCC): C22.0 | Cirrhosis: biopsy/radiology/ ascites or oesophageal varices + physician’s annotation of cirrhosis.  Varices: upper endoscopy or oesophageal varices treated with band ligation  Ascites: clinical examination or radiology  HCC: biopsy-proven or EASL guidelines | Unspecified: 121/133 = 91 (85-95)  Alcohol-related: 126/136 = 93 (87-96)  Varices: 118/123 = 96 (91-99)  HCC: 91/109 = 84 (75-90)  Ascites 56/129 = 43 (35-52)  Ascites+ code for chronic liver disease 50/54 = 93 (82-98) |
| Schollin, 2019 (26) | Nationwide  (1987-2013) | **Inpatient** | Intussusception in children <3 years of age | ICD-9: 560A | Inpatient register: K56.1 | Brighton collaboration clinical case definition | Definite: 330/392: 84 (82–86)  Probable 12/330= 3.6  Possible 6/392=1.5 |
| Jakobsson, 2017 (27) | Nationwide  (1987-) | **NPR** | Inflammatory bowel disease (IBD), Crohn’s disease (CD), ulcerative colitis (UC) | Individuals with ≥2 diagnoses (ICD-9), NPR: CD 555 or UC 556 | Individuals with ≥2 diagnoses, NPR: CD K50 or UC K51 | Definite diagnosis: Copenhagen criteria Probable diagnosis: physician’s judgement | Any IBD: 120/129=93 (87-97)  UC: 79 (66-88)  CD: 72 (60-82) |
| Östensson, 2023 (28) | Regional  (1997-2017) | **NPR** | Childhood-onset Inflammatory Bowel Disease | - | Children <18 years with ≥2 diagnoses, NPR:  CD K50  UC K51  K52.3 | ECCO-ESGAR Guideline | 57/61=93 (87-100) |
| Mouratidou, 2022 (29) | Nationwide  (2002-2014) | **NPR** | Childhood-onset Inflammatory Bowel Disease (IBD) | - | Children <18 years with ≥2 diagnoses, NPR:  CD K50  UC K51  IBD-U (UC + CD, or K52.3) | Copenhagen criteria and the revised Porto criteria | IBD: 216/233 = 93 (89-96)  CD 80/102 = 78  UC 65/88 = 74  IBDU 8/35 = 23 |
| Razavi, 2011 (30) | Nationwide  (1998, 2007) | **Inpatient** | Acute pancreatitis | **-** | Inpatient register: K85.0, K85.1, K85.2, K85.3, K85.8, K85.9 | Definite diagnosis: 2/3 of: (1) upper abdominal pain, (2) elevated amylase or lipase (3) typical signs on medical imaging | First diagnosis in 2007: definitive or probable 191/192 = 99  In 1998 181/182 = 99 |
| Munch, 2017 (31) | Multicenter  (2010-2012) | Inpatient | Incident acute pancreatitis (AP) and incident primary malignancy | - | Women with osteoporosis aged>55 years (post-menoposal) with    a main diagnosis in inpatient register K85    or a primary or secondary diagnosis in NPR: malignancy: C00xxa – C43xx, C45xx – C97xx | Incident acute pancreatitis (AP) and incident primary malignancy (IPM) | AP: 43/49 = 87.8 (75.8–94.3)  Malignancy: 32/40= 80.0 (65.2–89.5) |
| Åhsberg, 2011 (32) | Multi-center  (1993-1994, 1999-2000) | **Inpatient** | Peptic ulcer | Inpatient register (ICD9):  Bleeding gastric ulcer (GU); 531A, 531E, K25.0, K25.4,  Bleeding duodenal ulcer (DU); 532A, 532E K26.0, K26.4,  Bleeding gastroduodenal ulcer (without specified location); 533A, 533E, K27.0, K27.4,  Perforated gastric ulcer 531B, 531C, 531F, 531G, K25.1, K25.2, K25.5, K25.6,  Perforated duodenal ulcer 532B, 532C, 532F, 532G, K26.1, K26.2, K26.5, K26.6,  Perforated gastroduodenal ulcer 533B, 533C, 533F, 533G, K27.1, K27.2, K27.5, K27.6,  Unspecified (not bleeding, not perforated) gastric ulcer (UNS) 531D, 531H, 531X, K25.3, K25.7, K25.9,  Duodenal ulcer UNS 532D, 532H, 532X, K26.3, K26.7, K26.9  Gastroduodenal ulcer UNS 533D, 533H, 533X, K27.3, K27.7, K27.9 | Inpatient register:  Bleeding gastric ulcer (GU); 531A, 531E, K25.0, K25.4,  Bleeding duodenal ulcer (DU); 532A, 532E K26.0, K26.4,  Bleeding gastroduodenal ulcer (without specified location); 533A, 533E, K27.0, K27.4,  Perforated gastric ulcer 531B, 531C, 531F, 531G, K25.1, K25.2, K25.5, K25.6,  Perforated duodenal ulcer 532B, 532C, 532F, 532G, K26.1, K26.2, K26.5, K26.6,  Perforated gastroduodenal ulcer 533B, 533C, 533F, 533G, K27.1, K27.2, K27.5, K27.6,  Unspecified (not bleeding, not perforated) gastric ulcer (UNS) 531D, 531H, 531X, K25.3, K25.7, K25.9,  Duodenal ulcer UNS 532D, 532H, 532X, K26.3, K26.7, K26.9  Gastroduodenal ulcer UNS 533D, 533H, 533X, K27.3, K27.7, K27.9 | Misclassified bleeding ulcer a) operation for bleeding ulcer or b) symptoms/history and endoscopically verified ulcer c) an endoscopically verified bleeding ulcer. Misclassified perforated ulcer: operation for perforated ulcer/in another way verified perforated ulcer. | Misclassified bleeding ulcer 1993-1994: 65/190= 34.2  1999-2000: 98/197= 49.7 |
| **Rheumatology** |  |  |  |  |  |  |  |
| Wallman, 2022 (33) | Multi-center  (2003-2015) | **Outpatient** | Psoriatic arthritis | - | Outpatient register: L40.5, M07.0, M07.1, M07.2, M07.3 at rheumatology or internal medicine department | CASPAR, Moll and Wright, Vasey and Espinoza, or modified ESSG criteria for PsA. | Any criteria: 343/400=86  CASPAR 69  Moll and right 51  Vasey and Espinoza 76  ESGN: 64 |
| Ceder, 2021 (34) | Single-center  (2010-2013) | **NPR** | Sarkoidosis | - | Individuals with ≥2 diagnoses, NPR: D86 | Reviewer’s judgement | 94/100=94 (87-98)  Definite: 77  Probable 17 |
| Lindström, 2015 (35) | Nationwide  (2007-2009) | **Outpatient** | Ankylosing spondylitis (AS) and undifferentiated spondyloarthritis (uSpA) | - | Outpatient register: M460, M641, M468, M469 at rheumatology department | Modified New York (mNY), Assessment of SpondyloArthritis international Society, (ASAS), Amor, and European Spondyloarthropathy Study Group (ESSG) criteria | Any criteria: 222/250=89 (AS)  147/186=79 (uSpA)  mNY-criteria (AS): 70  ASAS citeria (AS): 79  mNY-criteria (uSpA): 20 |
| Raaschou, 2011 (36) | Nationwide  (1999-2005) |  | Patient with RA exposed and not exposed to biologics, diagnosed with breast, colorectal, lung, non-melanoma skin cancer or prostate cancer | Not reported |  | Not reported | RA diagnosis in biologics-treated: 81/82=99    Biologics-naïve: 73/76=96  Cancer diagnosis: 100 |
| Waldenlind, 2014 (37) | Single-center  (2005-2008) | **Outpatient** | Rheumatoid arthritis (RA) |  | Individuals with >1 diagnosis assigned by a rheumatologist (in >1 visit), outpatient register: M05.9, M06.0 | 2010 ACR/EULAR and the 1987 ACR-classification criteria for RA. | Prevalent patients: 91/100=91  Incident patients= 85/102=83 |
| Berthold, 2019 (38) | Regional (2002-2010) | **NPR** | Juvenile idiopathic arthritis (JIA) |  | Individuals <16 years, NPR: M08, M09 | 2001 ILAR classification | 307/484=63 |
| **Respitatory** |  |  |  |  |  |  |  |
| Inghammar, 2013 (39) | Multi-center (1990-2008) | Inpatient | Asthma, sarkoidosis, pneumoconiosis, pulmonary fibrosis, and bronchiectasis in adults with invasive pneumococcal disease and control subjects | Adults with invasive pneumococcal disease and control subjects, inpatient register:  Asthma  ICD-8/9 493  Sarkoidosis  ICD-8/9: 135  Pneumoconiosis  ICD-8: 515-516  ICD-9: 500, 501, 502. 503. 504, 505  Pulmonary fibrosis  ICD-8/9: 515, 516  Bronchiectasis  ICD-8/9: 494 | Adults with invasive pneumococcal disease and control subjects, inpatient register:  Asthma: J45, J46  Sarkoidosis: D86.0, D86.2, D86.8, D86.9  Pneumoconiosis: J60, J61, J62, J63, J64, J66  Pulmonary fibrosis: J84.0, J84.1, J84.8  Bronchiectasis: J47 | Not stated | Asthma: n=n=198=  80  Sarcoidosis: n=17=95  Pneumoconiosis: n=7=100  Pulmonary fibrosis: n=41=85  Bronchiectasis: n=13=85 |
| Örtqvist, 2013 (40) | Nationwide (2005-2009) | **NPR** | Pediatric asthma |  | NPR: J45 in individuals <18 years | Swedish Paediatric Society's section for Allergy, 2012. | PPV in NPR only:  0-4.5 years: 78  >4.5-17 years: 99 |
| Inghammar, 2012 (41) | Regional  (2002-2007) | Inpatient | Chronic obstructive pulmonary disease | Main or secondary diagnosis, inpatient register (ICD-9): 491, 492, 496 | Main or secondary diagnosis, inpatient register: J41, J42, J43, J44 | Proven (GOLD criteria), probable, possible uncertain, unlikely | Proven: 81/374=21.7  Proven, probable, possible, uncertain: 348/374=93 |
| **Dermatology** |  |  |  |  |  |  |  |
| Grönhagen, 2017 (42) | Multicenter (2001-2012) | **NPR** | Bullous pemphigoid |  | Patients >20 years of age, NPR: L12.0, L12.8, L12.9 | Histopathological records coded for SNOMED codes D3618 and D36180, immunopathological records and/or medical records | 283/307=92 |
| Albadri, 2023 (43) | Multicenter  (2005-2018) | **NPR** | Dermatitis hepatiformis |  | L13.0 as 1^st^ or 2^nd^ diagnosis | Granular IgA deposits in the papillary dermis using direct immunofluorescence OR 2 of the minor criteria according to S2k-guidelines 2021 | 90/144, corrected for missing data 100/160=62.5 |
| Zagoras, 2023 (44) | Single-center | Outpatient | Genodermatoses |  | Q80.0–Q80.9, Q81.0–Q81.9, Q82.8DEF, Q828H–Q82.8R and Q82.8W, Q828T, Q82.9, Q85.0, Q87, Q87.5 | Clinical descriptions, photographs, histopathology, family history and molecular genetic analyses | 96.1 (92.4–98.2)  Darier disease:90.9 (62.3–98.4) Porokeratosis 93.1 (86.2–96.8)  Ichthyosis EB, Hailey-Hailey disease Gorlin syndrome: 100 |
| Adelborg, 2017 (45) | Multicenter (2005-2014) | **NPR** | Dermatologic events and hypersensitivity leading |  | Postmenopausal women with osteoporosis with Dermatologic-related hospitalization or ER visit, NPR:  Dermatologic events  L10, L12, L13, L14, L50, L511, L512, L518, L519, L538, R21  Hypersensitivity  D721, M364, M022, T782, T886, T887, T784 | Reviewer’s judgement | Dermatologic events leading to hospitalization or ER visit 39/42=92.9 (81.0–97.5)  Hypersensitivity leading to hospitalization or ER visit 29/48=60.4 (46.3–73.0) |
| **Neurology** |  |  |  |  |  |  |  |
| Sundholm, 2017 (46) | Regional  (2006-2013) | **NPR** | Idiopathic intracranial hypertension (IIH) |  | NPR: G93.2 | Modified Dandy Criteria | 135/207=65 |
| Sundholm, 2018 (47) | Regional  (2006-2013) | **NPR** | Idiopathic intracranial hypertension (IIH) |  | NPR: G93.2 | Previously validated IIH cases against the modified Dandy Criteria | ≥2: 91.2 (81.6- 96.0)  ≥3: 91.2 (81.6- 96.0)  ≥5: 83.8 (73.0- 90.9) |
| Nägga, 2022 (48) | Region  (1991-2014) | **NPR** | Dementia  Alzheimer’s disease (AD), vascular dementia (VaD), Mixed (AD+VaD), Parkinson’s disease (PDD), dementia with Lewy bodies (DLB), frontotemporal dementia (FTD), unspecified dementia | AD: 331A/331.0  VaD: 290E/290.4  FTD: 331B/331  unspecified dementia: 290, 294B/294.1, 331 C/331.2 | AD: F00, G30  VaD: F01,  PDD: F023  DLB: F028, G318A  FTD: F020, G310,  unspecified dementia: F03 | DSM-V | Any dementia: 2120/2206=96  AD: 503/792=63.5  Mixed: 214/258=82.9  VaD: 390/540=72.2  DLB: 47/73=64.4  PDD: 39/49=79.6  FTD: 17/26=65.4  Unspecified dementia: 112/445=25.2 |
| Nilsson, 2016 (49) | Regional  (2002-2009) | **NPR** | Dementia  Alzheimers disease (AD), Vascular dementia (VaD), or mixed dementia | Not reported |  | DSM-IIIR for all-cause dementia, DSM-IV for AD and VaD | 428/471=90.9 |
| Longinetti, 2022 (50) | Nationwide (1991-2014) | **NPR** | Amyotrophic lateral sclerosis (ALS) | Patients with concurrent multiple sclerosis (MS), myasthenia gravis (MG), inflammatory polyneuropathies (IP), or dermatopolymyositis (DMPM), NPR:  ALS  ICD-9: 335.C  MS  ICD-9: 340  MG  ICD-9: 358  IP  ICD-9: 357  DMPM  ICD-9: 710D, 710E | Patients with concurrent multiple sclerosis (MS), myasthenia gravis (MG), inflammatory polyneuropathies (IP), or dermatopolymyositis (DMPM), NPR:  ALS: G12.2  MS: G35  MG: G70.0  IP: G61  DMPM: M33, G72.4, G73.7 | El-Escorial criteria | 151/242=62 |
| Teljas, 2021  (51) | Regional  (2001-2013) | **NPR** | Multiple sclerosis (MS) | NPR (ICD-9): 340 | NPR: G35.9 | Clinical onset symptoms, assessment by the neurologist, cerebrospinal fluid analysis (CSF), and magnetic resonance imaging (MRI) reports  with conclusive evidence of MS. | ≥1 ICD code: 763/805=95  Best balance of sensitivity and specificity: ≥3 visits. Best sensitivity and PPV: ≥1 hospitalization or ≥2 clinic visits in ≥ 3 years; and ≥2 visits in ≥3 years |
| Iacobaeus, 2018  (52) | Nationwide (1988-2013) | **Inpatient and CDR** | Progressive multifocal leukoencephalopathy (PML) | Inpatient register and CDR (ICD-9): 046D | Inpatient register and CDR: A81.2 | Consensus statement from the AAN neuroinfectious disease section, 2013 | Definite, probable, or possible:108/250=43  For patients diagnosed in neurology departments: 82 |
| Olsson, 2015  (53) | Single-center (1997-2011) | **NPR and Cancer register** | Craniopharyngiomas (CPs)  *Patients with a secreting pituitary adenoma diagnosis were excluded.* |  | ≥1 diagnosis from a neurosurgical/ neurological care unit OR ≥2 diagnosis from internal medicine/endocrine care unit in the NPR:  ICDO/3 C75.1 and C24+ histological code 881 in the Cancer register | Not specified | 83/86=97 |
| **Psychiatry** |  |  |  |  |  |  |  |
| Rautio, 2021 (54) | Nationwide (1998-2016) | **NPR** | Hypochondriasis and dysmorphophobia | - | F45.2, F452A | DSM-IV-TR, DSM V | Hypochondriasis: 67/84=80 (70-88)  Dysmorphophobia: 111/122=91 (84-95) |
| Kouppis, 2020  (55) | Regional  (1987-2005) | **NPR** | Prsonality disorders (PD)  Emotionally unstable personality disorder (EUPD) | PD  ICD-9: 3010, 3012, 3013, 3014, 3015, 3016, 3017, 3018, 3019  EUPD  ICD-9: 3013 | PD:  F600-609  EUPD: F600-609  F603 | ICD and DSM-IV-TR, DSM V | PD: 88/95=93 (ICD); 77/95=81 (DSM)  EUPD: Based on ICD 26/26=100; Based on DSM= 20/26=77 |
| Hollander, 2019 (56) | Multi-center (2013-2015) | **NPR** | Post-traumatic stress disorder (PTSD) |  | F43.1 | DSM-IV, DSM-5. Mini International Neuropsychiatric Interview (M.I.N.I.). | 158/187=84 (79-90)  (DSM-IV)  141/187=75 (69-82)  (DSM-5) |
| Rück, 2015 (57) | Nationwide (1969-2010) | **NPR** | Tic disorders  Obsessive-compulsive disorder (OCD) | Tic disorders  ICD-8: 306,2  ICD-9: 307C  OCD  ICD-8: 300,3  ICD-9: 300D | NPR:  Tic disorders: F95.X  OCD: F42.X | ICD-10 and DSM-IV-TR | Tic disorders  59/64 = 92 (82-97)  OCD: 49/68 = 72 (60-82)  Only ICD10:  Tic disorders: 97  OCD 91-96 |
| Vilaplana-Perez, 2020 (58) | Nationwide (1998-2016) | **NPR** | Social anxiety disorder |  | F40.1 | ICD-10 and DSM-IV-TR | 77/95 =81 (72-88) |
| Reutfors, 2010  (59) | Regional  (1984-2000) | **Inpatient** | Schizophrenia spectrum, i.e. schizophrenia, schizophreniform or schizoaffective disorder | ICD-8/9: 295 | F20, F25 | OPCRIT check list | 94/168=56 |
| Allebeck, 2023 (60) | Nationwide (1973-2011) | **Inpatient** | Schizophrenia in men born 1950–1952 conscripted for compulsory military training in 1969–1970 | ICD-8 (1970-1986) 295,00-295,99, 297,00-297,98, 298,00-298,99, 299,99  ICD-9 (1987-1996): 295 A-H,W,X, 297B-297X, 298A-X | F20-29 | OPCRIT check list | 158/187=84 |
| Sellgren, 2011  (61) | Regional  (1973-2004) | **Inpatient** | Bipolar disorder  *Those with ≥1 inpatient hospitalization due schizophrenia were excluded.* | Individuals with ≥2 hospital discharges, inpatient register:  ICD-8: 296.00, 296.1, 296.2, 296.3, 296.88, 296.99  ICD-9: 296.0, 296.1, 296.2, 296.3, 296.4, 296.8, 296.9 | Individuals with ≥2 hospital discharges, inpatient register: F30, F31 | DSM IV-TR | 110/135=81 |
| **Malignancy** |  |  |  |  |  |  |  |
| Abildgaard, 2023 (62) | Multi-center  (2010-2017) | **NPR+PDR** | Multiple myeloma |  | C90.0+prescribed medication | Not reported | 38/55=69 |
| Löfstedt, 2023 (63) | Nationwide  (1997-2011) | **NPR** | Malignancy-associated hemophagocytic lymphohistiocytosis |  | D76.0-D76.3 and C96.0 + C00-C97 | Clinical and lab data | 54/62=87 |
| **Other conditions** |  |  |  |  |  |  |  |
| Friberg, 2016 (64) | Regional  (2006-2013) | **NPR+ CDR** | Bleeding events in patients with atrial fibrillation | - | NPR: I60, I61, I62, S064, S065, S066, K226, K25 (subcodes 0,2,4,6 only), K26 (subcodes 0,2,4,6 only), K27 (subcodes 0,2,4,6 only), K28 (subcodes 0,2,4,6 only), K290, K625, K661, K920- K922, I850, I983, N02, R319, N939, N950, N501A, H113, H313, H356, H431, H450, H922, I312, J942, M250, R04, R58, T810, D500, D629 | Physician’s judgement | Any bleeding =94.1 (91.9-95.9)  Fatal bleeding: =88.1 (82.1-92.7) |
| Bergstrom, 2011 (65) | Regional  (2000-2004) | **Inpatient** | Injuries  *Following diagnoses were excluded: T36–T65, T96 and T97* | - | Inpatient register:  Adverse effects S00–T80  Poisoning (T78) | Judgement of experienced intensive care unit nurse, based on discharge summary, progress notes, x-ray results and nursing records | Principal and secondary injury diagnosis codes correct to the 3rd position: 791/967=81.8  External cause codes: 1299/1370=94.8 |

ESC, European Society of Medicine

# **Supplemenatry Table 4.** Sensitivity or positive predictive value (PPV) for variables in the National Patient Register (NPR), compared to other registers, cohorts, or databases

| **Author, year** | **Setting**  **(Observation)** | **NPR data part** | **Diagnosis/condition** | **Definition** | | **Reference standard** | **PPV (%)** | **Sensitivity (%)** |
| --- | --- | --- | --- | --- | --- | --- | --- | --- |
|  |  |  |  | **ICD-7/8/9** | **ICD-10** |  |  |  |
| **Quality/other registers** |  |  |  |  |  |  |  |  |
| Sakakibara, 2024 (66) | Nationwide  2018-2020 | NPR | Cancer | - | C00-C80 (excluding C43-C44) | Swedish Cancer Register | Lip, oral cavity, and pharynx: 87.5  Digestive organs: 95.6  Respiratory and intrathoracic organs: 91.3  Bone and articular cartilage: 47.8  Mesothelial and soft tissue: 40.5  Breast: 97.4  Female genital organs: 90.0  Male genital organs: 89.2  Urinary tract: 88.1  Eye, brain, and other parts of central nervous system: 88.4  Thyroid and other endocrine glands: 84.8  Ill-defined, secondary, and unspecified sites: 8.8 | 82.2  79.1  77.7  55.6  64.9  91.0  19.9  87.3  79.4  54.9  32.3  24.9 |
| Meyer, 2020 (67) | (2008-2017) | Inpatient register, | Hip fracture | ≥60 years of age (ICD-9), inpatient register: 820 | ≥60 years of age, inpatient register: S720-722;  NOMESCO: NFB and NFJ | Swedish Hip Fracture Register | Primary diagnosis: 114,292/140,724=81.2  Primary or secondary diagnosis: 115,785/144,857=79.9 |  |
| Bergdahl, 2021 (68) | Single-centre  (2011-2012) |  | Humeral fracture | - | Individuals ≥16 years, NPR: S42.2, S42.3, S42.4 | Swedish Fracture Register+NPR | 1513/2173=69.6 (67.7-71.6) | 1648/1699=97.0 |
| Birgegård, 2022 (69) | (2008-2013) |  | Eating disorders (anorexia nervosa (AN)  bulimia nervosa (BN)  Unspecified ED  ED not otherwise specified (EDNOS) | - | NPR ICD codes:  AN: F50.0  BN: F50.2  ED: unspecified F50.9 | National Quality Register for Eating Disorder Treatment (RiksÄt)  DSM IV | AN: 74.7  BN: 83.6  EDNOS: 76.1 | AN: 75.1  BN: 62.9  EDNOS: 82.7 |
| Murley, 2019 (70) | (2001-2013) |  | Multiple sclerosis (MS) | - | Main or contributory diagnosis, NPR: G35 | The Swedish MS Register (MSREG), Prescribed Drug Register, Cause of Death Register MiDAS, LISA | 18291/19781=92.5 |  |
| Köster, 2013 (71) | 2004 | NPR | Stroke | - | Individuals ≥20 years, inpatient register: I61, I63,I64, G45–46, I60, I62, I65–69, R96–99 | WHO stroke criteria in the Northern MONICA quality register | Definite or possible stroke: 1636/2032 80.5 (78.8-82.2) | Definite or possible stroke 1636/1832= 89.3(87.9-90.7) |
| Indremo, 2021  (72) | Nationwide (2006-2014) | NPR | Gender Dysphoria |  | NPR:  Transsexualism: F64.0  Other gender identity disorders: F64.8  Gender identity disorder, unspecified: F64.9 | **NPR:** Surgical gender confirming treatments: mastectomy and breast reductions (HAC10, HAC15, HAC20, HAC99, HAD20, HAD30, HAD35, HAD99, HAE99) and genital surgeries (KFH50, KGV30, KGW96, KGH96, LCD00, LCD01, LCD04, LCD10, LCD11, LCD96, LCD97, LED00); breast reconstruction (HAD00, HAD10, HAD99, HAE00, HAE20, HAE99), genital surgeries (LEE10, LEE40, LEE96, LFE10, LFE96 and KGC10) and larynx surgery (DQD40)  **PDR:** Gender confirming hormonal treatment: ATC codes: testosterone (G03B) antiandrogens and estrogen (G03C, L02AA, G03D, L02AB, G03H, L02BB, G04CB, C03DA01, L02AE, H01CA) and puberty blockers (L02AE, H01CA) | ≥ 1 diagnosis; 1408/2086=67  ≥4 diagnoses 1374/1739=79 |  |
| **Other cohorts** |  |  |  |  |  |  |  |  |
| Rizzuto, 2018 (73) | (1998-2002) |  | Dementia  Alzheimer's disease (AD)  Vascular dementia (VaD)  Other dementia (OD) | Inpatient register:  ICD-7: 304, 305, 306;  ICD-8: 290, 293.0, 293.1    ICD-9: 290.0, 290.1, 290.4, 290.8, 290.9, 294.1, 331.0, 331.1, 331.2, 331.9 | **NPR:** F00, F00.0, F00.1, F00.2, F00.9, F01, F01.0, F01.1, F01.2, F01.3, F01.8, F01.9, F02, F02.0, F02.1, F02.2, F02.3, F02.4, F02.8, F03, F03.9, F05.1, G30, G30.0, G30.1, G30.8, G30.8, G31.1, G31.8A | Multiple population-based study cohorts:  National: HARMONY, SATSA, OCTO-Twin, and GENDER;  Regional: KP, SNAC-K where diagnosis was based on DSMIII-R, structured interview, medical exam and psychological assessment. | Dementia:  81.3 (75.4–86.3)  AD:  56.6 (47.6 - 65.3),  VaD:  35.5 (19.2 - 54.6)  OD:  15.9 (8.24–26.7) | Dementia:  47.3 (44.1–50.5)  AD:  32.5 (28.9-36.4)  VaD:  11.6 (6.9-18.0)  OD:  24.9 (18.5–32.1) |
| Arkema, 2016 (74) | (1964-2009) | NPR | Systemic lupus erythematosus (SLE) | SLE ICD codes | SLE ICD codes | Verified cases from clinical cohorts established at four university hospitals | ≥2 visits, at least one at a specialist clinic: 80.1 (after accounting for sampling ) |  |
| Feldman, 2012 (75) | (1998-2004) | Inpatient | Parkinsonian disorder and Parkinson’s disease | ICD7: 350;  ICD8: 342.00, 342.08, 342.09  ICD9: 332.0, 332.0; | G20, G21.4, G21.8, G21.9, G23.1, G23.2, G23.9, G25.9 | The Screening Across the Lifespan Twin study (SALT) of twins born before 1958 identified 194 cases of parkinsonian disorders and 132 with Parkinson’s disease (gold standard) | Parkinsonian disorder: 66/75= 88 (78.4-94.4)  Parkinson’s disease  51/72=70.8 (58.9-81.0) | Parkinsonian disorder: 123/194= 63.4 (56.2-70.2)  Parkinson’s disease 96/132= 72.7(64.3-80.1) |
| Appelros, 2011 (76) | (1999-2000) | Inpatient | Stroke | ICD-10 codes I61, I63 and I64 |  | Community-based stroke register, using the WHO criteria |  | 318/357=89.1 (85.4, 92.1) |
| Dahl, 2019 (77) | 2014 | NPR | Lyme neuroborreliosis | ICD-10: A69.2 + G01.9 |  | Positive cerebrospinal fluid–serum anti-Borrelia antibody index, diagnosed at Karolinska University Hospital Laboratory |  | 67/150=44.7 (36.6, 53.0) |
| Baturova, 2014 (78) | (2001-2011) | NPR+CDR | Atrial fibrillation in patients with ischemic stroke | ICD-9: 427D | I48 | Electrocardiogram database, reviewed by a cardiologist | 152/188=81 | 152/190=80 |
| Norberg, 2013 (79) | Single-center  (2004-2010) | **NPR** | Atrial fibrillation | - | NPR: I48 | Marquette system for computerized interpretation of ECGs or other source of ECG or history of converted ECG in medical chart. | 2119/2196=96.5 | 2119/2274=93.2 |
| Bergman, 2015 (80) | Nationwide and single-center  (2005-2010) | **NPR +PDR** | Pediatric hemodynamically significant congenital heart disease |  | Algorithm based on age at diagnosis, diagnostic codes, surgical procedure codes, and dispensing records | Treatment with palivizumab in local hospital data | 27/34=79 (62-91) | 69/86=80 (70-88) |
| Hedman, 2018 (81) | National and regional (1992-2012) | **NPR (+PDR)** | Pediatric asthma |  | Twins 9-12 years born 1992-2012, NPR: J45, J46 + dispensing of asthma medications (in PDR) | 1) Parent-reported asthma in CATSS cohort questionnaire (cohort 1)  2) Doctor-reported diagnosis from specialist and primary care data from Stockholm (in cohort 2) | (calculated by reviewer)  Cohort 1:  PPV: 2003/2789=71.8  Cohort 2:  PPV: 282/506=55.7 | Cohort 1: 2003/2649=75.6  Cohort 2: 282/360=78.3 |
| Lundström, 2015 (82) | Nationwide (1993-2009) | **NPR** | Autism Spectrum Disorders (ASD) | Twins <10 years of age born 1993-2002, NPR (ICD-9): 299A | Twins <10 years of age born 1993-2002, NPR: F84.0, F84.1, F84.5, F84.9 | The Autism- Tics, ADHD and other Comorbidities inventory (parental telephone interview) |  | 50/190* (26,3, 20-33)  =51 (twins screened positive for ASD who in childhood had an ASD diagnosis recorded in NPR) |

(CDR: Cause of Death Register)

# **Supplementary Table 5.** Studies validating ATC and KVÅ-codes in the National Patient Register (NPR)

| **Author, year** | **Setting (observation)** | **Procedure type** | **Definition** | **Reference standard** | **PPV (%)** | **Sensitivity**  **(%)** |
| --- | --- | --- | --- | --- | --- | --- |
| Bröms, 2021 (83) | Multi-center (2005-2017) | ATC codes for biologic treatment in patients with inflammatory bowel disease | ATC (L04AA12) L04AB02, L04AB04, L04AB06, L04AA33, L04AC05 | Treatment episode in the patient chart |  | 127/1361= 9.3 |
| Shrestha, 2020 (84) | Multi-center (1999-2017) | Inflammatory bowel disease (IBD) subtype and phenotype | Algorithms based on ICD and/or KVÅ codes | Montreal classification in patient charts | Crohn’s disease:  L2: 36 (32-40)  L1L3LX: 81 (76-85)  B1: 61 (56-65)  B2/B3: 76 (72-81)  P: 83 (78-87)  Ulcerative colitis:  E1/E2:80 (71-89)  E3: 82 (78-87) |  |
| Forss, 2019 (85) | Nationwide (1966-2014) | Inflammatory bowel disease -related surgery | ICD-9: CD 555, UC 556 or ICD-10: CD K50, UC K51  60 IBD-related surgical procedures, categorized as abdominal resection, perianal procedures, and other surgery | Surgery recorded in patient chart | 153/155 = 98.7(96.3-100) | 155/164 = 94.5 (89.6–99.3) |
| Holmgren, 2018 (86) | Regional (2007-2013) | Stoma outcome after anterior resection for rectal cancer | KVÅ-codes for stoma reversal: JFG00, 10, 20, 23, 26, 29, 30, 33, 36 | Surgery recorded in patient chart | Stoma reversal: 99.1 (96.7–99.9)  Permanent stoma: 85.1 (75.8–91.8) |  |
| Tao, 2016 (87) | Nationwide (2011) | Obesity surgery  Gastric by-pass (GBP), other obesity surgery, non-obesity surgery | A majority of the codes starting with JDF or JFD." | Not reported | Obesity surgery overall: 555/572 = 97.0 (95.6-98.4)  GBP: 96.8 (95.1-98.5)  Other obesity surgery: 88.6 (83.8-93.4)  Non-obesity surgery: 78.3 (61.4–95.1) |  |
| Lagergren, 2016 (88) | Nationwide (1997-2010) | Date of surgery for esophageal cancer | Not reported | Operation charts for esophageal cancer surgery based on a nationwide cohort | Concordant date:  761/798 = 95.4 |  |
| Lagergren, 2012 (89) | Nationwide (1987-2005) | Oesophageal cancer resection surgery | ICD9: 150; ICD10: C15 and  282.X, 2821, 2822, 2829 before 1997 and codes JCC00, JCC10, JCC11, JCC20, JCC30, JCC96, JCC97 after 1997 **in Inpatient Register** | Operation charts from medical records | 1352/1358=99.6 |  |

# **References**

1. Molander V, Bower H, Askling J. Validation and characterization of venous thromboembolism diagnoses in the Swedish National Patient Register among patients with rheumatoid arthritis. Scandinavian journal of rheumatology. 2023;52(2):111-7.

2. Ohman L, Johansson M, Jansson JH, Lind M, Johansson L. Positive predictive value and misclassification of diagnosis of pulmonary embolism and deep vein thrombosis in Swedish patient registries. Clinical epidemiology. 2018;10:1215-21.

3. Andersson T, Isaksson A, Khalil H, Lapidus L, Carlberg B, Soderberg S. Validation of the Swedish National Inpatient Register for the diagnosis of pulmonary embolism in 2005. Pulm Circ. 2022;12(1):e12037.

4. Schaufelberger M, Ekestubbe S, Hultgren S, Persson H, Reimstad A, Schaufelberger M, et al. Validity of heart failure diagnoses made in 2000-2012 in western Sweden. ESC Heart Fail. 2020;7(1):36-45.

5. Basic C, Rosengren A, Lindstrom S, Schaufelberger M. High validity of cardiomyopathy diagnoses in western Sweden (1989-2009). ESC Heart Fail. 2018;5(2):233-40.

6. Emilsson L, Andersson B, Elfstrom P, Green PH, Ludvigsson JF. Risk of idiopathic dilated cardiomyopathy in 29 000 patients with celiac disease. J Am Heart Assoc. 2012;1(3):e001594.

7. Magnusson P, Palm A, Branden E, Morner S. Misclassification of hypertrophic cardiomyopathy: validation of diagnostic codes. Clinical epidemiology. 2017;9:403-10.

8. Gedeborg R, Holm L, Feltelius N, Sundström A, Eggers KM, Nurminen ML, et al. Validation of myocarditis diagnoses in the Swedish patient register for analyses of potential adverse reactions to COVID-19 vaccines. LID - 10.48101/ujms.v128.9290 [doi]. (2000-1967 (Electronic)).

9. Fedchenko M, Mandalenakis Z, Hultsberg-Olsson G, Dellborg H, Eriksson P, Dellborg M. Validation of myocardial infarction diagnosis in patients with congenital heart disease in Sweden. BMC Cardiovasc Disord. 2020;20(1):460.

10. Walas A, Svensson K, Gyris M, Bang P, Sundelin HEK. Paediatric ischaemic stroke is a valid diagnosis in the Swedish National Patient Register. Acta Paediatr. 2021;110(7):2179-86.

11. Ragnarsson O, Olsson DS, Chantzichristos D, Papakokkinou E, Dahlqvist P, Segerstedt E, et al. The incidence of Cushing's disease: a nationwide Swedish study. Pituitary. 2019;22(2):179-86.

12. Gkaniatsa E, Ekerstad E, Gavric M, Muth A, Trimpou P, Olsson DS, et al. Increasing Incidence of Primary Aldosteronism in Western Sweden During 3 Decades - Yet An Underdiagnosed Disorder. The Journal of clinical endocrinology and metabolism. 2021;106(9):e3603-e10.

13. Kamal W, Bjornsdottir S, Kampe O, Trolle Lagerros Y. Concordance Between ICD-10 Codes and Clinical Diagnosis of Hypoparathyroidism in Sweden. Clinical epidemiology. 2020;12:327-31.

14. Rasmark Roepke E, Christiansen OB, Hansson SR. Reliability of recurrent pregnancy loss diagnosis coding in the Swedish National Patient Register: a validation study. Clinical epidemiology. 2019;11:375-81.

15. Sward EM, Schriever TU, Franko MA, Bjorkman AC, Wilcke MK. The epidemiology of scaphoid fractures in Sweden: a nationwide registry study. J Hand Surg Eur Vol. 2019;44(7):697-701.

16. Tampe U, Frank S, Weiss RJ, Jansson KA. Diagnosis of Open Tibial Fracture Showed High Positive Predictive Value in the Swedish National Patient Register. Clinical epidemiology. 2020;12:1113-9.

17. Südow HA-O, Sjödin LA-O, Mellstrand Navarro CA-O. Validity of distal radius fracture diagnoses in the Swedish National Patient Register. (2047-783X (Electronic)).

18. Stalfors J, Enoksson F, Hermansson A, Hultcrantz M, Robinson A, Stenfeldt K, et al. National assessment of validity of coding of acute mastoiditis: a standardised reassessment of 1966 records. Clin Otolaryngol. 2013;38(2):130-5.

19. Bergdahl J, Jarnbring F, Ehrenstein V, Gammelager H, Granath F, Kieler H, et al. Evaluation of an algorithm ascertaining cases of osteonecrosis of the jaw in the Swedish National Patient Register. Clinical epidemiology. 2013;5:1-7.

20. Mahmood MW, Schmidt PA-O, Olén O, Hellsing C, Hjern F, Abraham-Nordling M. Identification of diverticular disease in Swedish healthcare registers: a validation study. (1502-7708 (Electronic)).

21. Tornkvist NT, Backman AS, Linder M, Altman M, Simren M, Olen O, et al. Identification of irritable bowel syndrome in the Swedish National Patient Register: a validation study. Scandinavian journal of gastroenterology. 2023;58(7):709-17.

22. Aberg F, Shang Y, Strandberg R, Wester A, Widman L, Hagstrom H. Four-fold increased mortality rate in patients with Wilson's disease: A population-based cohort study of 151 patients. United European gastroenterology journal. 2023;11(9):852-60.

23. Giunta DA-O, Karlsson PA-O, Younus M, Berglind IA, Kieler HA-O, Reutfors JA-O. Validation of diagnoses of liver disorders in users of systemic azole antifungal medication in Sweden. (1471-230X (Electronic)).

24. Astrom H, Wester A, Hagstrom H. Administrative coding for non-alcoholic fatty liver disease is accurate in Swedish patients. Scandinavian journal of gastroenterology. 2023;58(8):931-6.

25. Bengtsson B, Askling J, Ludvigsson JF, Hagstrom H. Validity of administrative codes associated with cirrhosis in Sweden. Scandinavian journal of gastroenterology. 2020;55(10):1205-10.

26. Schollin Ask L, Svensson JF, Olen O, Ortqvist A. Clinical presentation of intussusception in Swedish children under 3 years of age and the validity of diagnostic coding. Pediatr Surg Int. 2019;35(3):373-81.

27. Jakobsson GL, Sternegard E, Olen O, Myrelid P, Ljung R, Strid H, et al. Validating inflammatory bowel disease (IBD) in the Swedish National Patient Register and the Swedish Quality Register for IBD (SWIBREG). Scandinavian journal of gastroenterology. 2017;52(2):216-21.

28. Ostensson M, Bjorkqvist O, Guo A, Stordal K, Halfvarson J, Marild K, et al. Epidemiology, validation, and clinical characteristics of inflammatory bowel disease: the ABIS birth cohort study. BMC gastroenterology. 2023;23(1):199.

29. Mouratidou N, Malmborg P, Jaras J, Sigurdsson V, Sandstrom O, Fagerberg UL, et al. Identification of Childhood-Onset Inflammatory Bowel Disease in Swedish Healthcare Registers: A Validation Study. Clinical epidemiology. 2022;14:591-600.

30. Razavi D, Ljung R, Lu Y, Andren-Sandberg A, Lindblad M. Reliability of acute pancreatitis diagnosis coding in a National Patient Register: a validation study in Sweden. Pancreatology. 2011;11(5):525-32.

31. Munch T, Christensen LB, Adelborg K, Tell GS, Apalset EM, Westerlund A, et al. Positive predictive values of ICD-10 codes to identify incident acute pancreatitis and incident primary malignancy in the Scandinavian national patient registries among women with postmenopausal osteoporosis. Clinical epidemiology. 2017;9:411-9.

32. Ahsberg K, Ye W, Lu Y, Zheng Z, Stael von Holstein C. Hospitalisation of and mortality from bleeding peptic ulcer in Sweden: a nationwide time-trend analysis. Alimentary pharmacology & therapeutics. 2011;33(5):578-84.

33. Wallman JK, Alenius GM, Klingberg E, Sigurdardottir V, Wedren S, Exarchou S, et al. Validity of clinical psoriatic arthritis diagnoses made by rheumatologists in the Swedish National Patient Register. Scandinavian journal of rheumatology. 2023;52(4):374-84.

34. Ceder S, Rossides M, Kullberg S, Eklund A, Grunewald J, Arkema EV. Positive Predictive Value of Sarcoidosis Identified in an Administrative Healthcare Registry: A Validation Study. Epidemiology. 2021;32(3):444-7.

35. Lindstrom U, Exarchou S, Sigurdardottir V, Sundstrom B, Askling J, Eriksson JK, et al. Validity of ankylosing spondylitis and undifferentiated spondyloarthritis diagnoses in the Swedish National Patient Register. Scandinavian journal of rheumatology. 2015;44(5):369-76.

36. Raaschou P, Simard JF, Neovius M, Askling J, Anti-Rheumatic Therapy in Sweden Study G. Does cancer that occurs during or after anti-tumor necrosis factor therapy have a worse prognosis? A national assessment of overall and site-specific cancer survival in rheumatoid arthritis patients treated with biologic agents. Arthritis Rheum. 2011;63(7):1812-22.

37. Waldenlind K, Eriksson JK, Grewin B, Askling J. Validation of the rheumatoid arthritis diagnosis in the Swedish National Patient Register: a cohort study from Stockholm County. BMC Musculoskelet Disord. 2014;15:432.

38. Berthold E, Mansson B, Kahn R. Outcome in juvenile idiopathic arthritis: a population-based study from Sweden. Arthritis Res Ther. 2019;21(1):218.

39. Inghammar M, Engstrom G, Kahlmeter G, Ljungberg B, Lofdahl CG, Egesten A. Invasive pneumococcal disease in patients with an underlying pulmonary disorder. Clin Microbiol Infect. 2013;19(12):1148-54.

40. Ortqvist AK, Lundholm C, Wettermark B, Ludvigsson JF, Ye W, Almqvist C. Validation of asthma and eczema in population-based Swedish drug and patient registers. Pharmacoepidemiol Drug Saf. 2013;22(8):850-60.

41. Inghammar M, Engstrom G, Lofdahl CG, Egesten A. Validation of a COPD diagnosis from the Swedish Inpatient Registry. Scandinavian journal of public health. 2012;40(8):773-6.

42. Gronhagen C, Nilzen K, Seifert O, Thorslund K. Bullous Pemphigoid: Validation of the National Patient Register in Two Counties in Sweden, 2001 to 2012. Acta Derm Venereol. 2017;97(1):32-5.

43. Albadri Z, Al Bayati D, Häbel H, Jerkovic Gulin S, Grönhagen C, Seifert O. Incidence of Dermatitis Herpetiformis in Sweden 2005 to 2018: A Nationwide Retrospective Cohort Study. (1651-2057 (Electronic)).

44. Zagoras T, Inci R, Kantere D, Holmström P, Broström J, Gillstedt M, et al. Incidence and Prevalence of 73 Different Genodermatoses: A Nationwide Study in Sweden. (1651-2057 (Electronic)).

45. Adelborg K, Christensen LB, Munch T, Kahlert J, Trolle Lagerros Y, Tell GS, et al. Positive predictive values of International Classification of Diseases, 10th revision codes for dermatologic events and hypersensitivity leading to hospitalization or emergency room visit among women with postmenopausal osteoporosis in the Danish and Swedish national patient registries. Clinical epidemiology. 2017;9:179-84.

46. Sundholm A, Burkill S, Sveinsson O, Piehl F, Bahmanyar S, Nilsson Remahl AIM. Population-based incidence and clinical characteristics of idiopathic intracranial hypertension. Acta Neurol Scand. 2017;136(5):427-33.

47. Sundholm A, Burkill S, Bahmanyar S, Nilsson Remahl AIM. Improving identification of idiopathic intracranial hypertension patients in Swedish patient register. Acta Neurol Scand. 2018;137(3):341-6.

48. Nagga K, Bransvik V, Stomrud E, Melander O, Nilsson PM, Gustavsson AM, et al. Prevalence and Ascertainment of Dementia Cases in the Malmo Diet and Cancer Study. J Alzheimers Dis Rep. 2022;6(1):529-38.

49. Nilsson ED, Melander O, Elmstahl S, Lethagen E, Minthon L, Pihlsgard M, et al. Copeptin, a Marker of Vasopressin, Predicts Vascular Dementia but not Alzheimer's Disease. J Alzheimers Dis. 2016;52(3):1047-53.

50. Longinetti E, Sveinsson O, Press R, Ye W, Ingre C, Piehl F, et al. ALS patients with concurrent neuroinflammatory disorders; a nationwide clinical records study. Amyotroph Lateral Scler Frontotemporal Degener. 2022;23(3-4):209-19.

51. Teljas C, Bostrom I, Marrie RA, Landtblom AM, Manouchehrinia A, Hillert J, et al. Validating the diagnosis of multiple sclerosis using Swedish administrative data in Varmland County. Acta Neurol Scand. 2021;144(6):680-6.

52. Iacobaeus E, Burkill S, Bahmanyar S, Hakim R, Bystrom C, Fored M, et al. The national incidence of PML in Sweden, 1988-2013. Neurology. 2018;90(6):e498-e506.

53. Olsson DS, Andersson E, Bryngelsson IL, Nilsson AG, Johannsson G. Excess mortality and morbidity in patients with craniopharyngioma, especially in patients with childhood onset: a population-based study in Sweden. The Journal of clinical endocrinology and metabolism. 2015;100(2):467-74.

54. Rautio D, Vilaplana-Perez A, Gumpert M, Ivanov VZ, Linde J, Osterman S, et al. Validity and reliability of the diagnostic codes for hypochondriasis and dysmorphophobia in the Swedish National Patient Register: a retrospective chart review. BMJ open. 2021;11(12):e051853.

55. Kouppis E, Ekselius L. Validity of the personality disorder diagnosis in the Swedish National Patient Register. Acta Psychiatr Scand. 2020;141(5):432-8.

56. Hollander AC, Askegard K, Iddon-Escalante C, Holmes EA, Wicks S, Dalman C. Validation study of randomly selected cases of PTSD diagnoses identified in a Swedish regional database compared with medical records: is the validity sufficient for epidemiological research? BMJ open. 2019;9(12):e031964.

57. Ruck C, Larsson KJ, Lind K, Perez-Vigil A, Isomura K, Sariaslan A, et al. Validity and reliability of chronic tic disorder and obsessive-compulsive disorder diagnoses in the Swedish National Patient Register. BMJ open. 2015;5(6):e007520.

58. Vilaplana-Perez A, Isung J, Krig S, Vigerland S, Jolstedt M, Bjureberg J, et al. Validity and reliability of social anxiety disorder diagnoses in the Swedish National Patient Register. BMC Psychiatry. 2020;20(1):242.

59. Reutfors J, Bahmanyar S, Jonsson EG, Ekbom A, Nordstrom P, Brandt L, et al. Diagnostic profile and suicide risk in schizophrenia spectrum disorder. Schizophr Res. 2010;123(2-3):251-6.

60. Allebeck PA-O, Gunnarsson T, Lundin AA-O, Löfving S, Dal H, Zammit S. Does a history of cannabis use influence onset and course of schizophrenia? (1600-0447 (Electronic)).

61. Sellgren C, Landen M, Lichtenstein P, Hultman CM, Langstrom N. Validity of bipolar disorder hospital discharge diagnoses: file review and multiple register linkage in Sweden. Acta Psychiatr Scand. 2011;124(6):447-53.

62. Abildgaard N, Freilich J, Anttila P, Bent-Ennakhil N, Ma Y, Lassenius M, et al. Use of Linked Nordic Registries for Population Studies in Hematologic Cancers: The Case of Multiple Myeloma. (1179-1349 (Print)).

63. Löfstedt A, Jädersten M, Meeths M, Henter JA-O. Malignancy-associated hemophagocytic lymphohistiocytosis in Sweden: incidence, clinical characteristics, and survival. (1528-0020 (Electronic)).

64. Friberg L, Skeppholm M. Usefulness of Health Registers for detection of bleeding events in outcome studies. Thromb Haemost. 2016;116(6):1131-9.

65. Bergstrom MF, Byberg L, Melhus H, Michaelsson K, Gedeborg R. Extent and consequences of misclassified injury diagnoses in a national hospital discharge registry. Inj Prev. 2011;17(2):108-13.

66. Sakakibara SA-O, Pazzagli LA-O, Linder MA-O. Consistency between the National Patient Register and the Swedish Cancer Register. (1099-1557 (Electronic)).

67. Meyer AC, Hedstrom M, Modig K. The Swedish Hip Fracture Register and National Patient Register were valuable for research on hip fractures: comparison of two registers. Journal of clinical epidemiology. 2020;125:91-9.

68. Bergdahl C, Nilsson F, Wennergren D, Ekholm C, Moller M. Completeness in the Swedish Fracture Register and the Swedish National Patient Register: An Assessment of Humeral Fracture Registrations. Clinical epidemiology. 2021;13:325-33.

69. Birgegard A, Forsen Mantilla E, Dinkler L, Hedlund E, Savva A, Larsson H, et al. Validity of eating disorder diagnoses in the Swedish national patient register. J Psychiatr Res. 2022;150:227-30.

70. Murley C, Friberg E, Hillert J, Alexanderson K, Yang F. Validation of multiple sclerosis diagnoses in the Swedish National Patient Register. European journal of epidemiology. 2019;34(12):1161-9.

71. Koster M, Asplund K, Johansson A, Stegmayr B. Refinement of Swedish administrative registers to monitor stroke events on the national level. Neuroepidemiology. 2013;40(4):240-6.

72. Indremo M, White R, Frisell T, Cnattingius S, Skalkidou A, Isaksson J, et al. Validity of the Gender Dysphoria diagnosis and incidence trends in Sweden: a nationwide register study. Scientific reports. 2021;11(1):16168.

73. Rizzuto D, Feldman AL, Karlsson IK, Dahl Aslan AK, Gatz M, Pedersen NL. Detection of Dementia Cases in Two Swedish Health Registers: A Validation Study. J Alzheimers Dis. 2018;61(4):1301-10.

74. Arkema EV, Jonsen A, Ronnblom L, Svenungsson E, Sjowall C, Simard JF. Case definitions in Swedish register data to identify systemic lupus erythematosus. BMJ open. 2016;6(1):e007769.

75. Feldman AL, Johansson AL, Gatz M, Flensburg M, Petzinger GM, Widner H, et al. Accuracy and sensitivity of Parkinsonian disorder diagnoses in two Swedish national health registers. Neuroepidemiology. 2012;38(3):186-93.

76. Appelros P, Terent A. Validation of the Swedish inpatient and cause-of-death registers in the context of stroke. Acta Neurol Scand. 2011;123(4):289-93.

77. Dahl V, Wisell KT, Giske CG, Tegnell A, Wallensten A. Lyme neuroborreliosis epidemiology in Sweden 2010 to 2014: clinical microbiology laboratories are a better data source than the hospital discharge diagnosis register. Euro Surveill. 2019;24(20).

78. Baturova MA, Lindgren A, Carlson J, Shubik YV, Bertil Olsson S, Platonov PG. Atrial fibrillation in patients with ischaemic stroke in the Swedish national patient registers: how much do we miss? Europace : European pacing, arrhythmias, and cardiac electrophysiology : journal of the working groups on cardiac pacing, arrhythmias, and cardiac cellular electrophysiology of the European Society of Cardiology. 2014;16(12):1714-9.

79. Norberg J, Backstrom S, Jansson JH, Johansson L. Estimating the prevalence of atrial fibrillation in a general population using validated electronic health data. Clinical epidemiology. 2013;5:475-81.

80. Bergman G, Haerskjold A, Stensballe LG, Kieler H, Linder M. Children with hemodynamically significant congenital heart disease can be identified through population-based registers. Clinical epidemiology. 2015;7:119-27.

81. Hedman AM, Gong T, Lundholm C, Dahlen E, Ullemar V, Brew BK, et al. Agreement between asthma questionnaire and health care register data. Pharmacoepidemiol Drug Saf. 2018;27(10):1139-46.

82. Lundstrom S, Reichenberg A, Anckarsater H, Lichtenstein P, Gillberg C. Autism phenotype versus registered diagnosis in Swedish children: prevalence trends over 10 years in general population samples. BMJ (Clinical research ed). 2015;350:h1961.

83. Broms G, Soderling J, Sachs MC, Halfvarson J, group Ss, Myrelid P, et al. Capturing biologic treatment for IBD in the Swedish Prescribed Drug Register and the Swedish National Patient Register - a validation study. Scandinavian journal of gastroenterology. 2021;56(4):410-21.

84. Shrestha S, Olen O, Eriksson C, Everhov AH, Myrelid P, Visuri I, et al. The use of ICD codes to identify IBD subtypes and phenotypes of the Montreal classification in the Swedish National Patient Register. Scandinavian journal of gastroenterology. 2020;55(4):430-5.

85. Forss A, Myrelid P, Olen O, Everhov AH, Nordenvall C, Halfvarson J, et al. Validating surgical procedure codes for inflammatory bowel disease in the Swedish National Patient Register. BMC medical informatics and decision making. 2019;19(1):217.

86. Holmgren K, Haapamaki MM, Matthiessen P, Rutegard J, Rutegard M. Anterior resection for rectal cancer in Sweden: validation of a registry-based method to determine long-term stoma outcome. Acta oncologica (Stockholm, Sweden). 2018;57(12):1631-8.

87. Tao W, Holmberg D, Naslund E, Naslund I, Mattsson F, Lagergren J, et al. Validation of Obesity Surgery Data in the Swedish National Patient Registry and Scandinavian Obesity Registry (SOReg). Obes Surg. 2016;26(8):1750-6.

88. Lagergren F, Mattsson F, Lagergren J. Validation of the date of surgery for esophageal cancer in the Swedish patient registry. Acta oncologica (Stockholm, Sweden). 2016;55(7):925-6.

89. Lagergren K, Derogar M. Validation of oesophageal cancer surgery data in the Swedish Patient Registry. Acta oncologica (Stockholm, Sweden). 2012;51(1):65-8.
